# *K*-operator for Modelling Neurodegeneration: Simulations, fMRI Application, Eigenvalue Analysis and Recurrence Plots

**DOI:** 10.1007/s10916-025-02244-6

**Published:** 2025-10-27

**Authors:** Sofia Fazio, Patrizia Ribino, Francesca Gasparini, Norbert Marwan, Peppino Fazio, Marco Gherardi, Maria Mannone

**Affiliations:** 1 Dipartimento di Fisica, Università Statale di Milano, Milano, Italy; 2 ICAR, National Research Council of Italy (CNR), Palermo, Italy; 3 Dipartimento di Informatica, Sistemistica e Comunicazione, Università degli Studi di Milano-Bicocca, Milano, Italy; 4 NeuroMI, Milan Center for Neuroscience, Milano, Italy; 5 Research Department Complexity Science, Potsdam Institute for Climate Impact Research (PIK), Member of the Leibniz Association, Potsdam, Germany; 6 Institute of Physics and Astronomy, University of Potsdam, Potsdam, Germany; 7 Institute of Geosciences Potsdam, University of Potsdam, Potsdam, Germany; 8 Dipartimento di Scienze Molecolari e Nanosistemi (DSMN), Ca’ Foscari University of Venice, Venezia, Italy; 9 Department of Telecommunications, VSB - Technical University of Ostrava, Ostrava, Czechia

**Keywords:** Functional network, *K*-operator, Alzheimer-Perusini’s disease progression, Parkinson, predictive models

## Abstract

The brain network damage provoked by a neurological disease can be modelled as the result of the action of an operator, *K*, acting on the brain, inspired by physics. Here, we explore the matrix formulation of *K*, analysing eigenvalues and eigenvectors, with heuristic considerations on different techniques to approximate it. The primary objective of this paper is to lay the foundational groundwork for an innovative framework aimed at the development of predictive models regarding the progression of neurodegenerative diseases. This endeavour will leverage the potential of integrating these novel representations of brain damage with advanced machine-learning techniques. A case study based on real-world data is presented here to support the proposed modelling.

## Introduction

Reading the mind has been mankind’s dream for centuries. Bridging the gap between physiology and thinking is a related question. Understanding anatomic structures and physiological processes inside the brain is still an ongoing adventure. The association between blood movements and thinking was an intuition of Angelo Mosso [1], who was working with open-skull patients. An intuition that led to the first rough but pioneering system of non-invasive neuroimaging. In 1890, he developed the so-called *human circulation balance*, a technique that could non-invasively measure the redistribution of blood during emotional and intellectual activity. His experiments provided foundational concepts for modern neuroimaging techniques, highlighting critical variables such as the *signal-to-noise ratio*, the appropriate choice of the experimental paradigm, and the need for simultaneous recordings of physiological parameters. Understanding the brain processes can also shed light on the mechanisms leading to diseases.

Being inspired by non-invasive studies on the brain, in this research we explore a recently-proposed mathematical operator to model neurological disorders [2], the so-called *K*-operator. In particular, the work proposed in this paper explores in detail a mathematical operator computed from the connectivity matrices of the brain functional network, objects with an important potential both in the fields of neurology and artificial intelligence. The proposed study starts with the investigation of two computational techniques to evaluate the action of the *K*-operator on the connectivity matrices.

Mainly, the first technique adopts the matrix product for the matrix inversion and the element-wise product for the computation of *K*. On the contrary, the second technique adopts the usual matrix product for the matrix inversion and *K* computation. The analogies between the two different resulting objects have been analysed, with a focus on their eigenvalues and eigenvectors. The rationale for adopting these two different computational techniques lies in their different informational outputs. The first technique provides easily interpretable data regarding which connections between pairs of brain areas are more damaged. In contrast, the second provides precise and cumulative information on the amount of damage.

A case study based on real-world data has been incorporated into this analysis. The data analysis conducted in the real case study underscores the similarities in the information generated by the two distinct techniques.

Hence, the primary objective of this study is to develop and compare different computational approaches for constructing the *K*-operator. These methods offer complementary perspectives on modelling disease effects on the brain connectome, and their comparison provides valuable insights into the operator’s behaviour and robustness. For each computational approach, we conduct a detailed analysis of the eigenvalues and eigenvectors of the resulting matrices, which helps characterise possible symmetry between different computational methods.

Thus, rather than aiming for immediate clinical generalisation or large-scale predictive modelling, we focus on establishing a theoretical and computational framework. To demonstrate its potential applicability, we illustrate the operator’s behaviour through synthetic examples and detailed analyses of individual clinical cases. This foundational approach lays the groundwork for future studies involving larger cohorts and integration with machine learning techniques, ultimately aiming to support diagnosis and prognosis in neurodegenerative diseases.

This research is the extension of the article *A physics-based view of brain-network alteration in neurological disease*, by the same authors, presented at the workshop Health Care at AIxIA (HC@AIxIA), in Bolzano in 2024 [3].

While the original paper introduced the basic concept and preliminary results, the current version incorporates several new contributions that deepen the mathematical foundation, expand the computational analysis, and enhance the real-data applications. The main new points addressed in this study are summarised below:

- Extended Mathematical Definition: Introduced a new, formal mathematical definition of the *K*-operator to clarify and generalise the original framework.
- Larger Simulated Data: Added simulations with 100×100 matrices, significantly expanding the scale of the computational experiments.
- Third Definition of the *K*-operator: Proposed an additional definition based on purely element-wise product, showing that eigenvalue patterns remain consistent with previous definitions, after a preliminary test in [4].
- Contrast with Kernel Product: Compared the element-wise product definition with the kernel product, observing different behaviours, thereby enriching the operator’s interpretation.
- Analysis of Artificial Dynamics: Evaluated the *K*-operator’s capacity to distinguish between various synthetic brain dynamics—null, increasing, decreasing, and varying models—confirming the usefulness of the *K*-operator to distinguish between them.
- New Real-Data Case Extension: Expanded the real-world analysis of Alzheimer-Perusini’s disease, including one additional time point to track disease progression.
- Region-Specific Recurrence Analysis: Performed a recurrence plot comparison focused on two selected brain regions, offering deeper insights into localised functional deterioration patterns.

The article is organised as follows. In “Related Works” section, we summarise the literature; in “Formalism and Methods” section, we describe the formalism; in “Toy Examples of Application” section, we propose some examples of applications on small matrices. In “A First Comparison of Eigenvalues for Real Data” section, we discuss a real-data case of Parkinson’s disease. Larger simulated matrices, and a third kind of product, are explored in “Extending the Analysis to Larger Matrices, and Adding a Third Definition of *K*” section. Then, *K* is exploited to detect dynamics within artificial data (“Using *K* to Detect Dynamics in Simulated Data” section), and to select brain regions worthy of interest from a case study of Alzheimer-Perusini’s disease (“An AD Case Study, Focusing on a Pair of ROIs” section). The Discussions (“Discussion” section), Limitations (“Limitations” section), and Conclusions (“Conclusions” section) end the article.

## Related Works

For decades, we viewed neurodegenerative disease anatomy through oversimplified frameworks. The beginning of human brain mapping in the late 1980s made it possible, through statistical methods, to determine disease topographies *in vivo* with network-based representations [1]. Non-invasive and computational methods for modelling the connectivity across the whole brain helped understand the alterations in the brain-network architecture [5]. Neuroimaging techniques play a crucial role in the study of neurodegenerative diseases. According to Seeley [6], two key concepts that still lead to many open questions are the onset and progression of these diseases, which have been investigated through "the study of *epicenters* whose connectivity in health mirrors the spatial patterning of each syndrome." In light of the studies by Royer and co-authors, atypical hub organisation in epilepsy and seizure activity are linked [5]. A network reorganisation is also occurring in Alzheimer-Perusini’s disease [7]. Using graph theory to study brain networks enables the calculation of topological parameters and the identification of network hubs [8]; quantifying them using centrality measures allows for investigations of relative differences between different types of epileptic patients and normal controls at the nodal level. Several studies also explored the value of network neuroscience approaches to provide clinically relevant measures in epilepsy, to develop the ability to capture seizures and investigate transient changes in network properties during the generation and evolution of seizures.

Studies on the connectome can also help bridge research in graph-theoretical frameworks with the latest development in machine learning. Attention to the activity of specific areas of the brain, which can be investigated in a certain detail in fMRI (Functional Magnetic Resonance Imaging), can also help relate neural imaging with other systems to investigate neurodegeneration, for example, speech patterns by machine learning, especially for Parkinson’s disease [9].

In a recent study, Mannone and co-authors [2] represented the damage to the brain network caused by some neurological diseases using a physics-inspired mathematical operator, the *Krankheit*-operator, in short *K*-operator. When applied to a diseased brain, *K* describes the progression of the disease over time. The authors also denoted the brain network as a block matrix $\mathcal {G}$, where each diagonal block represents the connections between brain lobes, while the off-diagonal blocks indicate the inter-lobe connections. In a different study, the authors applied this formalism specifically to Alzheimer-Perusini’s diseased brains [10].

Summarising, previous works on the *K*-operator concern the first, theoretical definition [2]; a first application to real data of healthy controls and Parkinsonian patients [11]; an application to Alzheimer-Perusini’s patients [10] and its development, with a multi-layer perceptron for prediction of disease progress [12]; an adaptation of the operator to electrocorticography (ECoG) data to investigate epilepsy [4]. The latest developments concern the definition of a space of brain states, where each point is a brain configuration as a *state*, and the transformation of a brain over time, be it within a normal or a pathological process, is described as a path within such a *brain space*, as a space of phases [13]. The present article is the development of a first analysis of the different definitions of the *K*-operator, compared in light of patterns of eigenvalues and eigenvectors [3]. Here, we consider an additional, complete case study with real data, and use *K* to select pairs of regions of interest of the brain to further analyse the corresponding time series with recurrence plots.

## Formalism and Methods

### The *K*-operator

Let us denote a healthy brain as a block matrix $\mathcal {G}$, where the blocks on the diagonal represent the connections inside the same lobe, and the others stand for the inter-lobe links. The damage provoked by a brain-based neurological disease can be modelled as the action of the *K*-operator:

1 $$\begin{aligned} K\mathcal {G} = \mathcal {G}^k, \end{aligned}$$

where the apex *k* is a label, and $\mathcal {G}^{k}$ is the matrix of a brain characterised by disease manifestations [2]. For different diseases, there will be different matrix elements of *K*. A possible choice for $\mathcal {G}$ is the matrix of weights of the connections between brain hubs. For instance, these matrices can be interpreted as functional connectivity matrices derived from resting-state functional Magnetic Resonance Imaging (rs-fMRI) data. These matrices represent the pairwise statistical dependencies—typically in the form of correlations or partial correlations—between the time series of neural activity recorded from different brain regions. Each entry in the matrix quantifies the strength of functional connectivity between a pair of regions of interest (ROIs), offering a network-based view of brain function at rest [10, 11].

We first analyse the action of the *K*-operator through the computation of $\mathcal {G}^k$ with two different product methods:

2 $$\begin{aligned} K*\mathcal {G} \qquad K@\mathcal {G} \end{aligned}$$

The first one is *Hadamard product*, also known as *element-wise product*, indicated here as $*$, and the second one is the *matrix product*, also known as *row-by-column-wise product*, indicated here as @. In the rest of the article, we will also use these symbols as upper indices to distinguish between the action of *K* computed through the *element-wise* product from the *K* computed through the *row-by-column-wise* product.

When $\mathcal {G},\,\mathcal {G}^k$ are known, how can we compute *K*? We can use a matrix inversion and an appropriate choice of the matrix product, one formally justified (*row-by-column*), the other yielding a similar sparsity of the results (*element-wise*). We want to investigate the similarities between these operators.

In the following, we show how *K* can act in simple cases and how it can be computed through the operation of matrix inversion. We will also analyse its mathematical properties. We consider two different kinds of products here to allow a more intuitive understanding of the action of *K*.

### Analysis of the *K*-operator with Classic Tools of Matrix Algebra

Let us denote with *A*, *B*, *C*, and *D* the elements of a $4\times 4$ *K*-operator. Let a toy $\mathcal {G}$ be defined as another $4\times 4$ matrix, having as diagonal elements (1-element blocks in this initial example) the connectivity inside the frontal lobe *f*, seen as a whole block, and *c*, the cerebellum, also seen as a whole block. The off-diagonal elements, also 1-element blocks, are the connectivity between *f* and *c*. In a real connectivity matrix, they are equal. If we consider the signal transmission from one area to another, we can separate the pathways according to the direction so that we can distinguish between $f\rightarrow c$ and $c\rightarrow f$. The *K* acting row-by-column is defined as follows:

3 $$\begin{aligned} K{@}\mathcal {G}= \left( \begin{matrix} A & B\\ C & D \\ \end{matrix}\right) {@} \left( \begin{matrix} f & fc \\ cf & c \\ \end{matrix}\right) =\left( \begin{matrix} Af + Bcf & Afc + Bc \\ Cf +Dcf & Cfc + Dc \\ \end{matrix}\right) . \end{aligned}$$

For the sake of simplicity, let us indicate the inverse of $\mathcal {G}$ as:

4 $$\begin{aligned} \mathcal {G}^{-1} = \left( \begin{matrix} m & n \\ o & p \\ \end{matrix} \right) \end{aligned}$$

Using the classical product, and imposing $fc = cf = x$, $K^{@}$ can be obtained as:

5 $$\begin{aligned} K^{@}= & \mathcal {G}^k\mathcal {G}^{-1} = \left( \begin{matrix} f^k & x^k \\ x^k & c^k \\ \end{matrix} \right) @ \left( \begin{matrix} m & n \\ o & p \\ \end{matrix} \right) \\& = \left( \begin{matrix} f^km + x^ko & f^kn + x^kp \\ x^km + c^ko & x^kn + c^kp \\ \end{matrix} \right) . \end{aligned}$$

### Formal Relationship Between *K* Computed Through Two Different Products in a Simple Case

If we consider *K* as a matrix of multiplying factors of the corresponding elements of the brain matrix, its element-wise action can be described as:

6 $$\begin{aligned} K{*}\mathcal {G}= \left( \begin{matrix} A' & B'\\ C' & D' \\ \end{matrix}\right) {*} \left( \begin{matrix} f & fc \\ cf & c \\ \end{matrix}\right) =\left( \begin{matrix} A'f & B'fc \\ C'cf & D'c \\ \end{matrix}\right) . \end{aligned}$$

Keeping the idea of the matrix inversion but using the element-wise product, we can develop a mixed technique that yields a symmetric and more easily interpretable form of the *K*-operator, as follows:

7 $$\begin{aligned} K^{*}=\mathcal {G}^k\mathcal {G}^{-1} = \left( \begin{matrix} f^k & x^k \\ x^k & c^k \\ \end{matrix} \right) *\left( \begin{matrix} m & n \\ o & p \\ \end{matrix} \right) = \left( \begin{matrix} f^km & x^kn \\ x^ko & c^kp \end{matrix} \right) . \end{aligned}$$

Although we can obtain the *K*-operator through two different mathematical methods, thanks to the theory of diagonalisation of matrices, we can notice some similarities between the computed operators, see “A First Comparison of Eigenvalues for Real Data” section. Analysing the mathematical properties, such as their eigenvalues and eigenvectors, we can justify the empirical evidence we can see through the simple visualisation of *K*.

We can establish a relationship between $K^{*}$ and $K^{@}$, defining a suitable *T*-matrix, such that:

8 $$\begin{aligned} K^{@}= & K^{*} + T = \left( \begin{matrix} f^km & x^kn \\ x^ko & c^kp \end{matrix} \right) \nonumber \\ & + \left( \begin{matrix} x^ko & f^kn+x^kp-x^kn \\ x^km + c^ko -x^ko & x^kn \end{matrix} \right) . \end{aligned}$$

### Eigenvalues and Eigenvectors

In the context of this first symbolic computation, we can rewrite (8) as:

9 $$\begin{aligned} K^{@} = K^{*}+T = \left( \begin{matrix} a & b \\ c & d \end{matrix} \right) + \left( \begin{matrix} e & f \\ g & h \end{matrix} \right) , \end{aligned}$$

which yields the eigenvalues:^1^

10 $$\begin{aligned} & \lambda _{1,2} = \frac{1}{2}\left[ \mp \right.\\&\left. \sqrt{(-a - d - e - h)^2 - 4 (a d + a h - b c - b g - c f + d e + e h - f g)} \right.\\&\left. + a + d + e + h\right] \end{aligned}$$

Neglecting the second-order elements depending on parts of the *T*-matrix, highlighting in bold the residual contribution of *T*, and rearranging the terms, the equation reads:

11 $$\begin{aligned} \lambda _{1,2} \sim \frac{1}{2}\left[ \mp \sqrt{(-a - d)^2 - 4 (a d - b c)} + (a + d)\right] + \frac{1}{2}\left( \textbf{e} + \textbf{h}\right) . \end{aligned}$$

Thus, the difference between the eigenvalues of *K* computed with the two techniques is mostly weighted by the *e* and *h* (i.e., $x^ko$ and $x^kn$, respectively), the anti-diagonal elements of $K^{*}$.

Concerning the *K*-operators obtained with the two different methods, we can think of getting $K^{@}$ from $K^{*}$ with some perturbation that we called *T*, see Eq. 8. *Perturbation theory* for matrices and linear algebra means estimating the change in the solution to a linear algebra problem caused by a small change in the input [14]. In this case, we can see the difference between the *K*-operators obtained with the two techniques as a perturbation; this allows us to see one technique as the perturbation of the other.

A detailed study of the perturbation-based approach is out of the scope of this article. In “Toy Examples of Application” section, we propose a sequence of examples to apply in different ways the formalism of the *K*-operator to some arbitrarily-defined matrices.

## Toy Examples of Application

In this section, we analyse some toy examples. In the first one, we will see the action of the *K*-operator on a healthy brain, modelled in four lobes, where each lobe is composed of 4 *hubs*, each one described by a $4\times 4$ matrix. In the second one, we will see the same action of the *K*-operator on a healthy brain, but with a simpler and more little model. On the other hand, in the last example, we will see how to obtain *K* from an inverse operation on the healthy and corrupted networks. In this analysis, we propose some formal considerations and a qualitative evaluation of the patterns of eigenvalues belonging to matrices obtained with different computational methods.

### Given $\mathcal {G}$ and *K*, find $\mathcal {G}^k$: Four Lobes, Four *hubs*

**Fig. 1:**
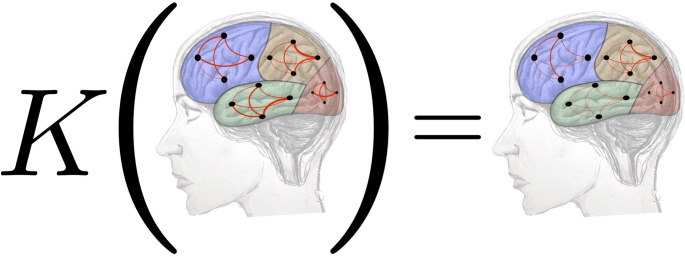
Action of *K* on a $16\times 16$ simplified brain (neglecting inter-lobe links as in [2]). Drawing by M. Mannone, graphs by S. Fazio

For a first experimental analysis, let us consider a model representing the healthy brain as a matrix. The *K*-operator is the action of a disease, while the output is a matrix of the diseased brain. Our healthy matrix is a diagonal block matrix, thus a square matrix $16\times 16$ such that the main diagonal blocks are square matrices, namely *A*, *B*, *C*, *D* in Eq. 12, each one a $4\times 4$ matrix, with all the off-diagonal blocks as 0-matrices, see Fig. 1. Also, the compromised-brain matrix is a diagonal block because *K* is shaped to act only on the diagonal blocks; this operator is defined according to [2]. The action of *K* is pictorially represented in Fig. 1.

For our purposes, we define symmetric matrices with 1 along the diagonal to have undirected graphs and the same weights from the *j*-th node to the *i*-th and vice versa. Then, we define the *K*-operator according to [2], and we compute the matrices related to the diseased brain first applying an element-wise product ($K *\mathcal {G}$) and then using the *matrix multiplication*, computed row by column ($K@ \mathcal {G}$). The first experimental analysis applied to these product matrices is the computation of their eigenvalues and eigenvectors. From this method, we could infer some analogies between the two techniques, above all among their eigenvalues. The eigenvalues and eigenvectors of the main matrix are simply those of the diagonal blocks combined. It means that when we have the eigenvalues and eigenvectors of $A^{k}$, $B^{k}$, $C^{k}$, $D^{k}$, we can also get the ones of the main matrix $16\times 16$ $\mathcal {G}^k$ representing the whole diseased brain, see Eq. 12.

12 $$\begin{aligned} & K\mathcal {G}|_{\text{ no } \text{ inter-lobe } \text{ submatrices }} \\& =K \left( \begin{matrix} A & & & \\ & B & & \\ & & C & \\ & & & D \end{matrix} \right) = \left( \begin{matrix} A^k & & & \\ & B^k & & \\ & & C^k & \\ & & & D^k \end{matrix} \right) \end{aligned}$$

**Fig. 2:**
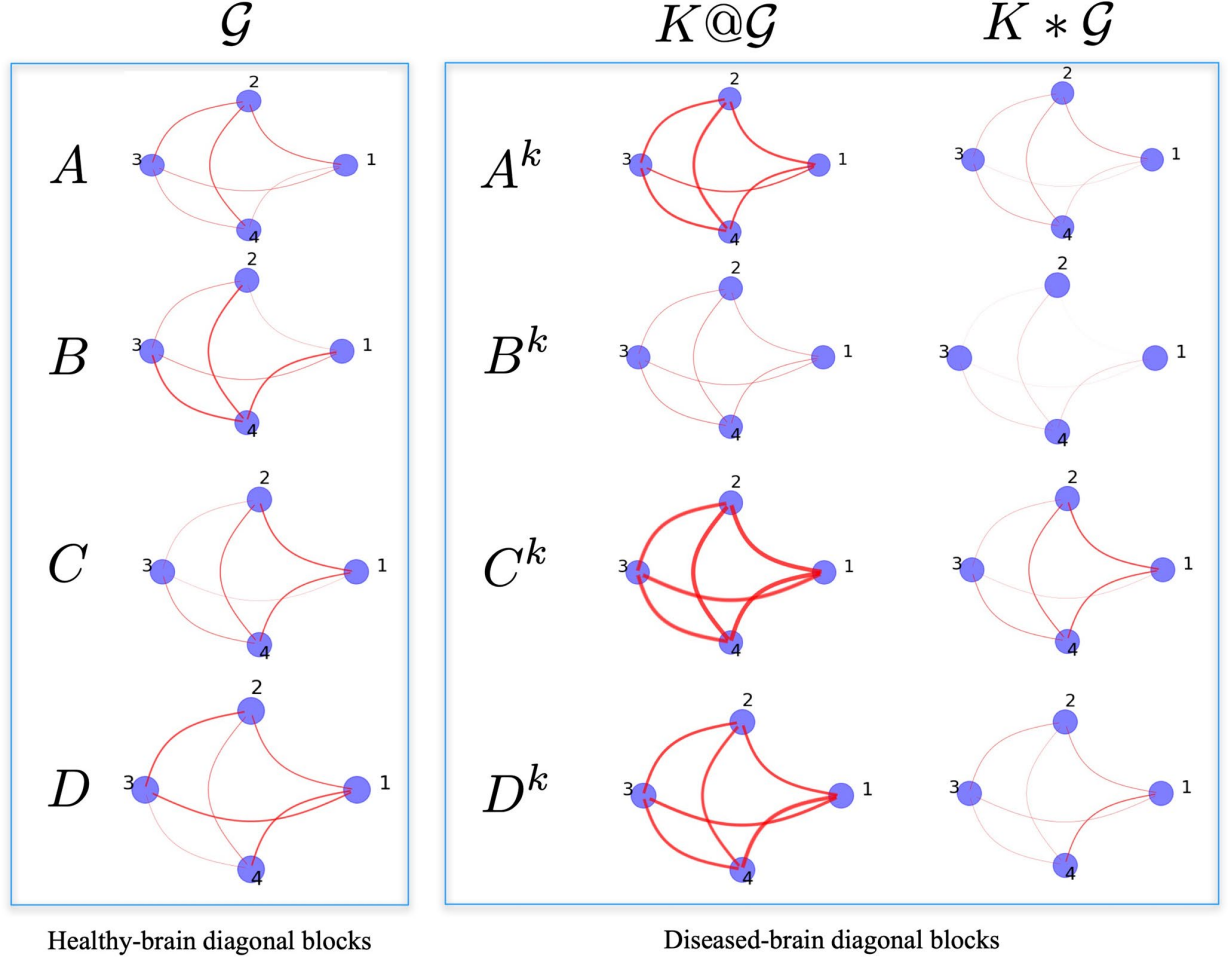
Graphs representing the intra-lobe connections of the healthy brain and of the compromised brain after the application of the *K*-operator, both with the row-by-column matrix multiplication (@) and the element-wise product ($*$)

The healthy-brain (sub)matrices are defined with entries’ values $\in [0,1]$. Up to a rescaling to $[-1, 1]$, this is coherent with the connectivity matrices and the connections from the brains’ real data [11]. Our choice is shown in Fig. 2 left. Having this knowledge, we should also remember that the matrices that we find through the element-wise products will still have entries between 0 and 1. On the other hand, matrices found with the row-by-column matrix multiplication will admit several entries with values exceeding 1. So, the defined matrices are biologically plausible, considering a normalisation. Another noteworthy element is that if we apply a symmetric *K* to a symmetric $\mathcal {G}$, in the element-wise computation case, we still get a symmetric $\mathcal {G}^{k}$; while if we get an asymmetric *K* acting row-by-column, and obtain a diseased-brain matrix, we notice that the weights between the *j*-th and *i*-th and between the *j*-th and *i*-th elements (intra-lobe connections) of the matrix are not the same.

The results of the row-by-column and element-wise action of *K*, yielding the diseased brain (sub)matrices, are shown in Fig. 2 center and right, respectively.

### Given $\mathcal {G}$ and *K*, Find $\mathcal {G}^k$: Four Lobes, One Hub Per Lobe

**Fig. 3:**
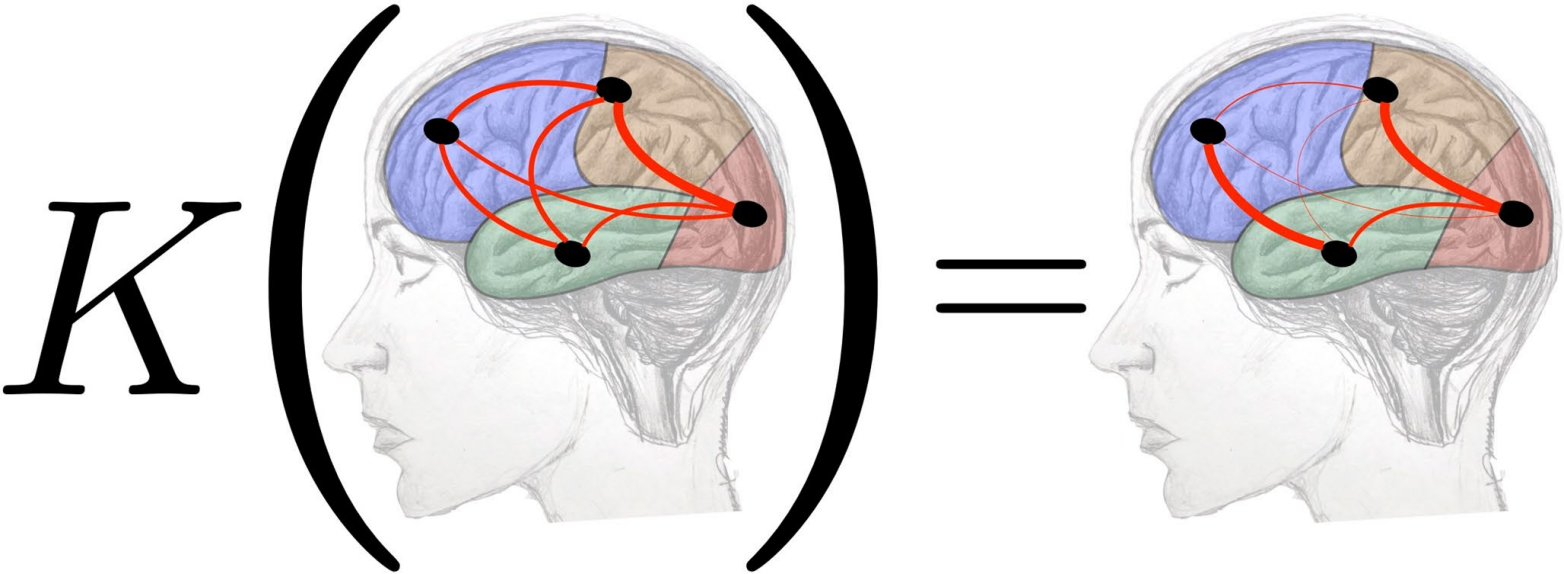
Action of *K* on a $4\times 4$ simplified brain. Drawing by M. Mannone, graphs by S. Fazio

Let us consider here the case of four lobes, with one hub per lobe, as shown in Fig. 3.

**Fig. 4:**
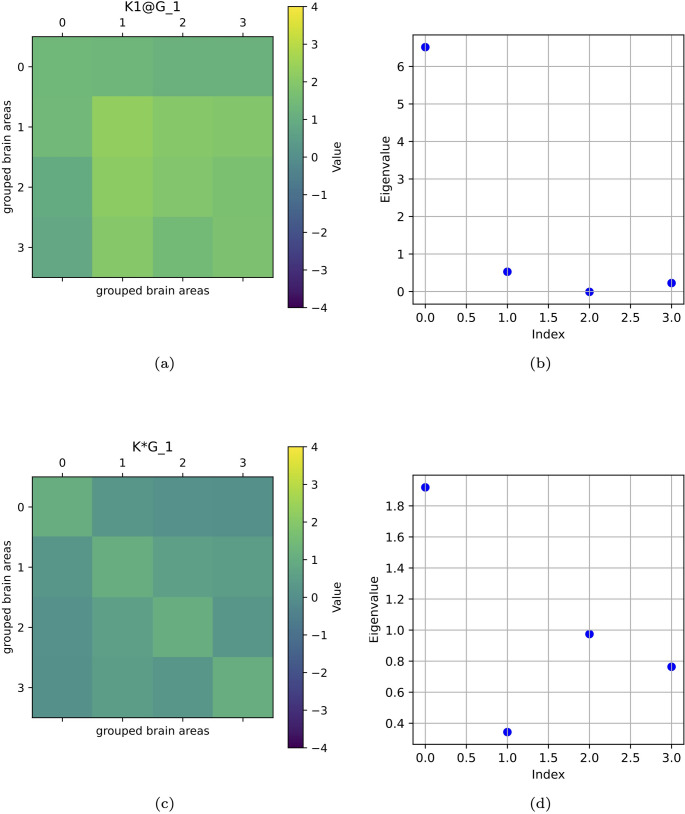
(a) *K*@*G* and eigenvalues (b), $K*G$ (c) and their eigenvalues (d). We notice a vertical correspondence between (a) and (c) and the higher value of the first eigenvalue (b, d)

As a further example, we choose a different shape of $\mathcal {G}$ and of *K* to compute $\mathcal {G}^k$ according to the diverse techniques (Fig. 4). Here, a $4\times 4$ matrix represents the whole brain to have a more suitable example of application of the theory presented in “Eigenvalues and Eigenvectors” section, yet simplified with respect to the example of “[Sec Sec9]” section. Darker pixels are in the first column-first row and vice versa, for the results obtained with both techniques. Also, the first eigenvalue is the highest in both cases. Here, we can also see some analogies, above all, among the eigenvalues of the different matrices, even if we chose a simpler model.

### Given $\mathcal {G}$ and $\mathcal {G}^k$, Find *K*

**Fig. 5:**
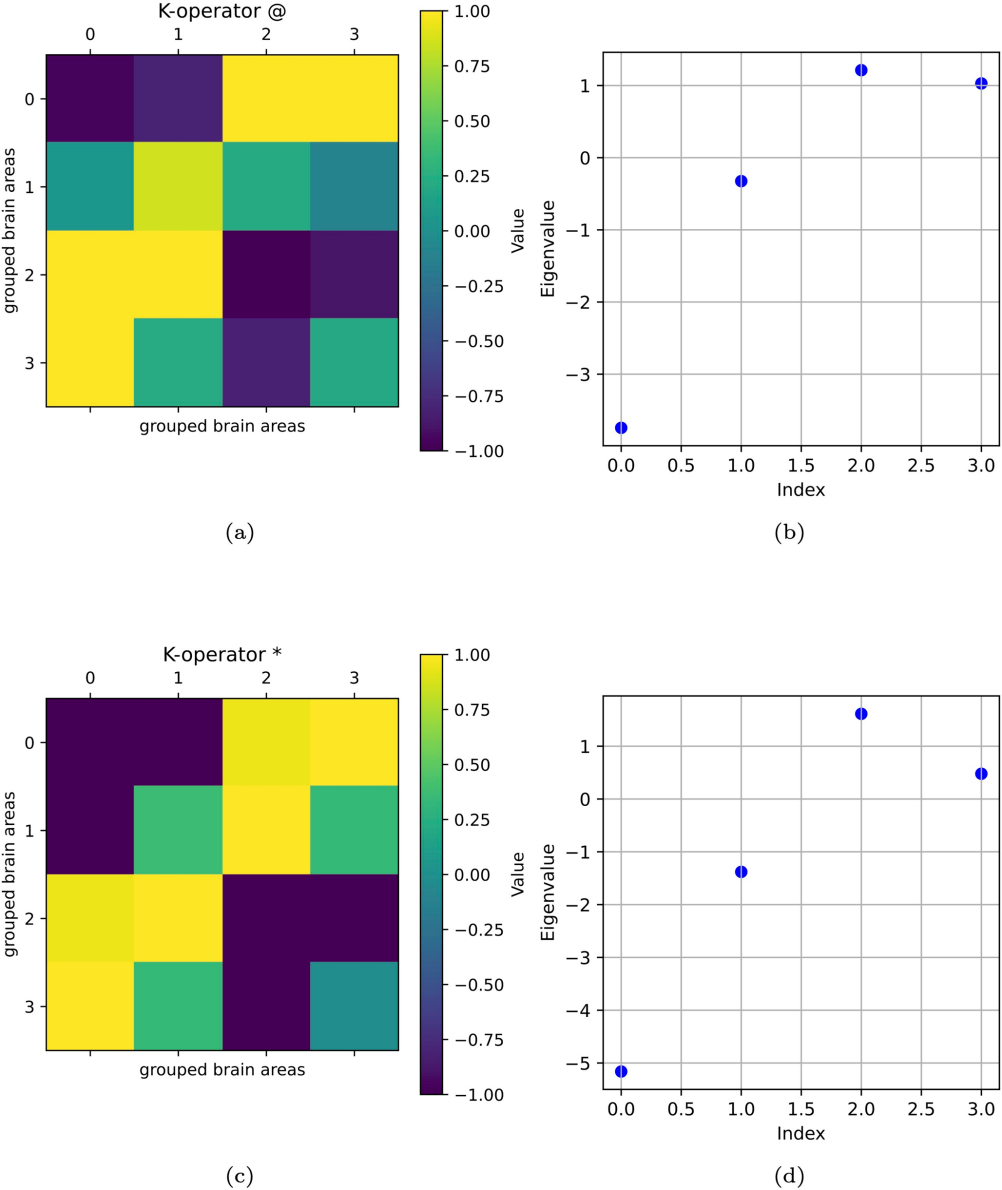
Two instances of *K*-operator visualised as heat-maps, computed rows-by-columns (a) and element-wise (c), and the corresponding eigenvalues (b, d)

**Fig. 6:**
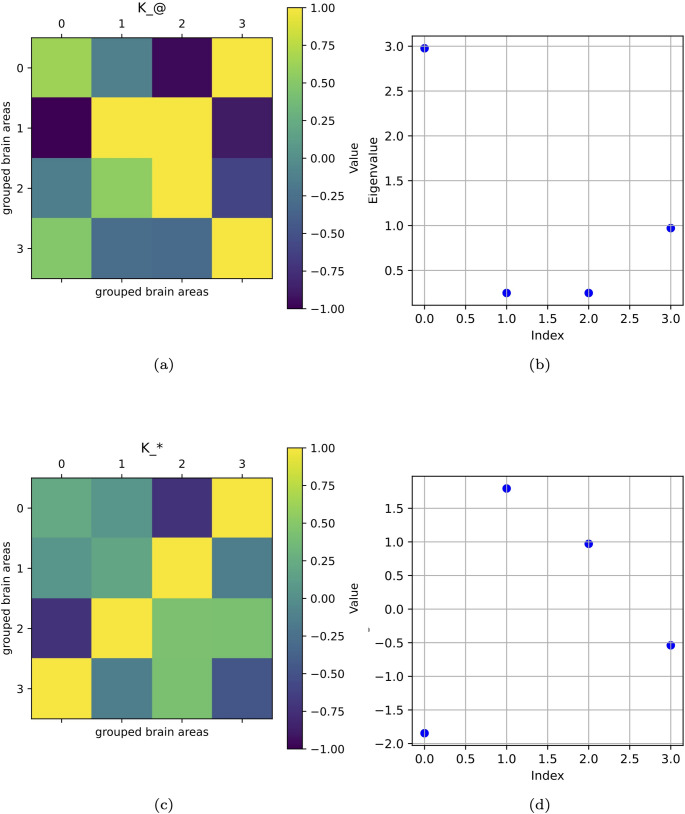
Other instances of *K*-operator, rows-by-columns (a) and element-wise (c), and the corresponding eigenvalues (b, d)

In “[Sec Sec9]” section, we defined an example of a simplified healthy brain matrix and an example of the *K*-operator, computing the diseased brain matrix according to two different kinds of product. In this section, we suppose to know the healthy brain matrix and the diseased one, and we compute *K*. We exploit the trick of matrix inversion, and compute $K^{@}$ according to classic algebra for a simple $2\times 2$ matrix (“Analysis of the *K*-operator with Classic Tools of Matrix Algebra” section). Then, we compare the $K^{@}$ with $K^{*}$, obtained through the element-wise product (“Formal Relationship Between *K* Computed Through Two Different Products in a Simple Case” section), and finally, we compute eigenvalues and eigenvectors of the two *K*s for a $4\times 4$ brain matrix (“Eigenvalues and Eigenvectors” section), and we discuss the possible connections between them. We compare instances of *K* (Figs. 5 and 6).

The scales of visualisations of eigenvalues are different because, given the different definitions of *K*, also the numerical values were different, and consequently, the numerical values of the eigenvalues. To highlight the similarity of patterns, we thus had to consider different scales, magnifying the visualisation for the smaller values. In fact, to highlight trend similarity, we needed different scales for the eigenvalue representation.

From the study of eigenvalues and eigenvectors belonging to the *K*-operator obtained with the two considered methods, a possible symmetry between the operators can emerge. Above all, eigenvalues mark some analogies between the different operators.

Our study is the first detailed and eigenvalue-based exploration of the possible analogies between the *K*-operators acting or computed through two different techniques. For this reason, we focused on eigenvalues because they allow us to extract information concerning the matrices. So, we collected our observations and we noticed some correspondences and regularities. Further research will aim to develop a theory of a generalised *K*-operator, and, from the algebraic point of view, theoretically justifying the correspondences we observed.

## A First Comparison of Eigenvalues for Real Data

We can apply the reasoning to one of the patients considered in previous studies, commenting on eigenvalues and eigenvectors of the *K*-operator derived for real data. We consider a female patient, 56 years old at the baseline, affected by Parkinson’s disease [11]. *K* is derived from the connectivity matrices at the baseline and follow-up, obtained from the resting-state functional magnetic resonance rsfMRI_RL, see Fig. 8. There are different analysis and processing tools for fMRI brain imaging data, see for example [15–17]. For our purposes, fMRI measurements are the starting point for the definition of instances of the *K*-operator. In particular, we considered the fMRI collected at the baseline and at the first follow-up. We downloaded the corresponding DICOM (Digital Imaging and Communications in Medicine) folder from the PPMI dataset (Parkinson’s Progression Markers Initiative),^2^ from which we derived the NIfTI file (Neuroimaging Informatics Technology Initiative). Choosing a parcellation of the brain (division into regions of interest), we finally extracted the time series for each region. From the time series for a set of fMRI, we computed the connectivity matrix. Let us now provide some further information on parcellation and connectivity matrices.

The **parcellation** is performed through the choice of an atlas. Among the possible brain atlases, we chose the Automated Anatomical Labelling (AAL3) for the detail provided in limbic and subcortical regions. A detailed analysis of the results that can be obtained with this method in the case of Parkinson’s Disease and their medical meaning is discussed in [11]. However, it is out of the scope of this article.

A **connectivity matrix** contains an estimation of the strength of the connections between brain regions. Using different atlases, we get different connectivity matrices. They are the subject of numerous studies that analyse them in detail [18]. However, we are here studying a mathematical object derived from them, the *K*-operator.

**Fig. 7:**
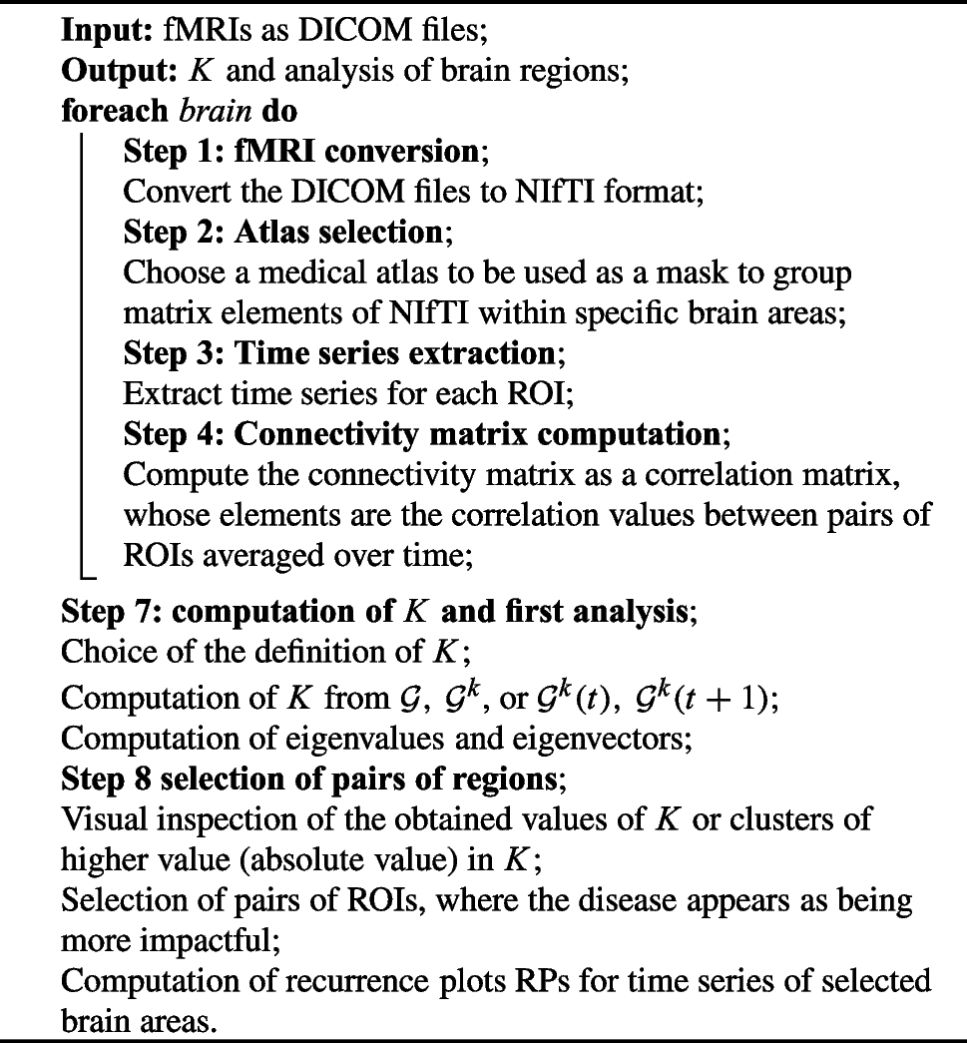
Brain matrices, *K*-operator, and analysis of pairs of regions

The pseudocode in the Algorithm of Fig. 7, adapted from [13], describes the workflow to obtain the brain matrices, the *K*-operator, and the analysis of pairs of brain regions.

**Fig. 8:**
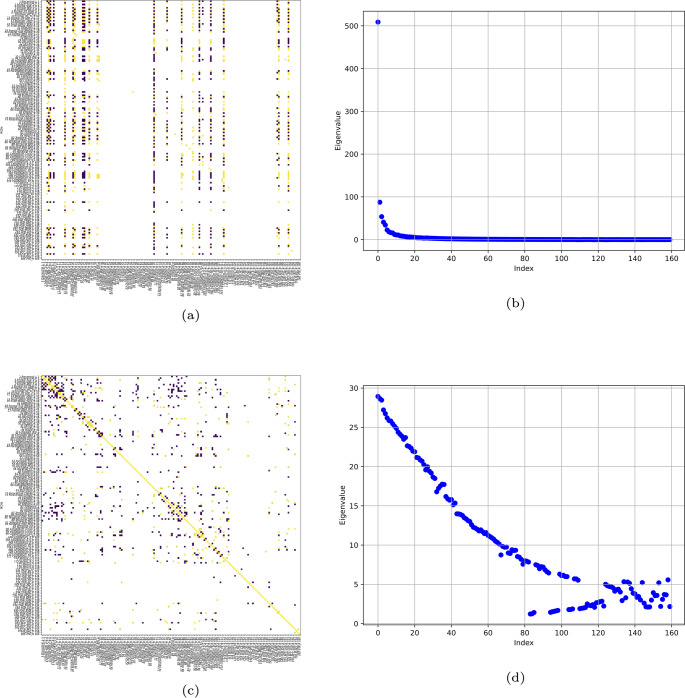
*K* computed from real data of a Parkinson’s disease female patient (atlas AAL3), with row-by-column (a) and element-wise product (c), and the respective eigenvalues (b,d). In (a), the elements above 12 are shown; in (c), above 0.8. (a) and (c) are taken from [11]

From a pair of connectivity matrices, considering them as the brain matrices characterizing the functional network at two-time points, we compute the *K*-operator. Our specific example is derived from [11]. We notice that the first eigenvalues are higher, also in the case of a larger matrix and real data, as it can be seen from Fig. 8. This confirms our observation for small matrices of synthetic data, considered in the previous Sections. In addition, observing the two *K* matrices (a, c) in Fig. 8, it seems that the first was cut and partially mirrored, to yield the symmetry of the second.

We can see some similarities, especially concerning the eigenvalues, aside from the computation technique adopted to find *K*. In particular, we notice the prevalence of the first eigenvalues for both techniques. Nevertheless, the scarceness of data regarding a full trend of Parkinson’s or Alzheimer-Perusini’s disruption [7], above all about the first stages of the disease, prevents us from investigating the detail of the mathematical properties of the *K*-operator, which will be the object of future studies.

### Computational Complexity Analysis

In this section, we analyze the computational complexity of the main procedures involved in our framework (see Algorithm 1). The complexity is expressed in terms of the size *n* of the brain network, i.e., the number of ROIs considered.

#### Functional Connectivity Matrix Computation

In our implementation, the functional connectivity matrix is computed using the numpy.corrcoef() Python function. This function calculates the Pearson correlation coefficients between all pairs of time series, given an input matrix:

13 $$\begin{aligned} X\in \mathbb {R}^{T \times n} \end{aligned}$$

where *T* is the number of time points and *n* is the number of ROIs. The output is an $n\times n$ symmetric correlation matrix *C*, where each entry $C_{ij}$ quantifies the linear dependence between the fMRI time series of ROI *i* and ROI *j*. Internally, numpy.corrcoef() function performs a series of optimised, vectorised operations to compute the correlation matrix efficiently. The function calculates the pairwise covariances by computing the dot products between all combinations of ROI time series. This step, which dominates the overall cost, has a complexity of $\mathcal {O}(T n^2)$. Finally, to transform the covariance matrix into a correlation matrix, each entry is normalised by the product of the standard deviations of the corresponding ROIs. This normalisation requires computing the standard deviation for each ROI and adjusting all matrix entries accordingly, resulting in an additional computational cost of $\mathcal {O}(Tn+n^2)$. Hence, the dominant cost arises from the covariance step, which requires computing pairwise dot products between all ROI pairs, leading to a total computational complexity of $\mathcal {O}(Tn^2)$. This means the computation scales quadratically with the number of ROIs and linearly with the number of time points.

#### Matrix Operations

A central step in our framework involves the application of the *K*-operator, which is defined in two forms. In its general formulation, the operator is computed via row-by-column-wise product (matrix product) according to Eq. 5 as follows:

14 $$\begin{aligned} K^{@}=\mathcal {G}^k\mathcal {G}^{-1} = \mathcal {G}^k@\mathcal {G}^{-1} \quad \text {where} \quad \mathcal {G}, \mathcal {G}^{-1} \in \mathbb {R}^{n \times n} \end{aligned}$$

The computational cost of performing this operation is dominated by the two matrix multiplications, which are carried out using numpy.matmul() Python function, requiring $\mathcal {O}(n^3)$ operations in the standard dense matrix case. Although more advanced algorithms for matrix multiplication exist (e.g., Strassen’s algorithm), they are generally not employed in standard scientific computing environments for matrices of moderate size and do not offer significant practical speedups in our setting.

An alternative version of the *K*-operator is defined via the Hadamard (element-wise) product according to Eq. 7 as follows:

15 $$\begin{aligned} K^{*}=\mathcal {G}^k*\mathcal {G}^{-1}\quad \text {where} \quad \mathcal {G}, \mathcal {G}^{-1} \in \mathbb {R}^{n \times n} \end{aligned}$$

In this case, each entry is computed independently, resulting in a total complexity of $\mathcal {O}(n^2)$, which is substantially more efficient.

#### Spectral Analysis

To characterise the behaviour of the *K*-operator, we systematically analyze the transformed matrices’ spectral properties by computing their eigenvalues. In our implementation, this is performed using the numpy.linalg.eig() Python function, which internally relies on routines optimised for dense matrices with a worst-case complexity of $\mathcal {O}(n^3)$. Consequently, eigenvalue computation along with matrix operations are the most computationally intensive steps in our framework.

#### Overall Computational Cost

Considering the main components of the framework, the dominant computational cost for a single application of the method can be expressed as:

16 $$\begin{aligned} \mathcal {O}(n^3) \end{aligned}$$

In experimental settings where the analysis is repeated across a number R of subjects, the overall cost scales linearly with R, yielding a total complexity of:

17 $$\begin{aligned} \mathcal {O}(R n^3) \end{aligned}$$

Finally, it is worth noting that in our experimental setup, the real connectome matrices are derived from standard brain atlases widely used in neuroimaging research, such as the Harvard-Oxford, MSDL, and AAL 3 atlases, all of which are available through the nilearn Python library. These atlases typically yield a number of ROIs ranging from approximately 70 to 171. For instance, the AAL 3 atlas defines 170 cortical and subcortical regions, while the MSDL atlas offers 39 functionally defined ROIs, and the Harvard-Oxford atlas provides around 96. Given this moderate matrix size (i.e., $n<=120$) along with typical fMRI time series lengths (e.g., $T\approx 1000$), and considering that all matrix operations in our framework (including matrix multiplication and eigenvalue decomposition) are performed using optimised linear algebra routines, the computational cost remains well within tractable limits. Consequently, the approach remains computationally feasible even when applied repeatedly across a large number of subjects, using standard scientific computing hardware.

## Extending the Analysis to Larger Matrices, and Adding a Third Definition of *K*

In previous results, we found vertical correspondence in the product matrices and similarities in the trend of their eigenvalues. The analogy between two or more matrices’ eigenvalues, referred to in algebra as *spectral similarity*, could imply that certain behaviours, like oscillation frequencies in dynamical systems and stability properties, are presumably alike. An important question to be addressed could be whether the comparisons we did and the correspondences we found can be carried out for larger matrices. In this section, we will investigate more deeply the action of the Krankheit-Operator in the case of bigger matrices representing the brain, thus applying it to bigger matrices than the toy examples in “Toy Examples of Application” section, namely $100\times 100$ matrices, considering a larger number of brain regions. Some of the results we are showing below have been obtained as part of the MSc in Physics of the first author [19].

We want to find the properties underlying this operator and go further in its analysis. This is easier and more accurate if we examine more parts of the brain. Afterwards, we will see other real-life data as well. As in the previous section, we are working with squared, non-singular matrices with entries between 0 and 1, while the real-data matrices contain values normalised between $-1$ and 1. In these cases, the analysis of eigenvalues and eigenvectors can provide insight into the properties and structure of the matrices.

**Fig. 9:**
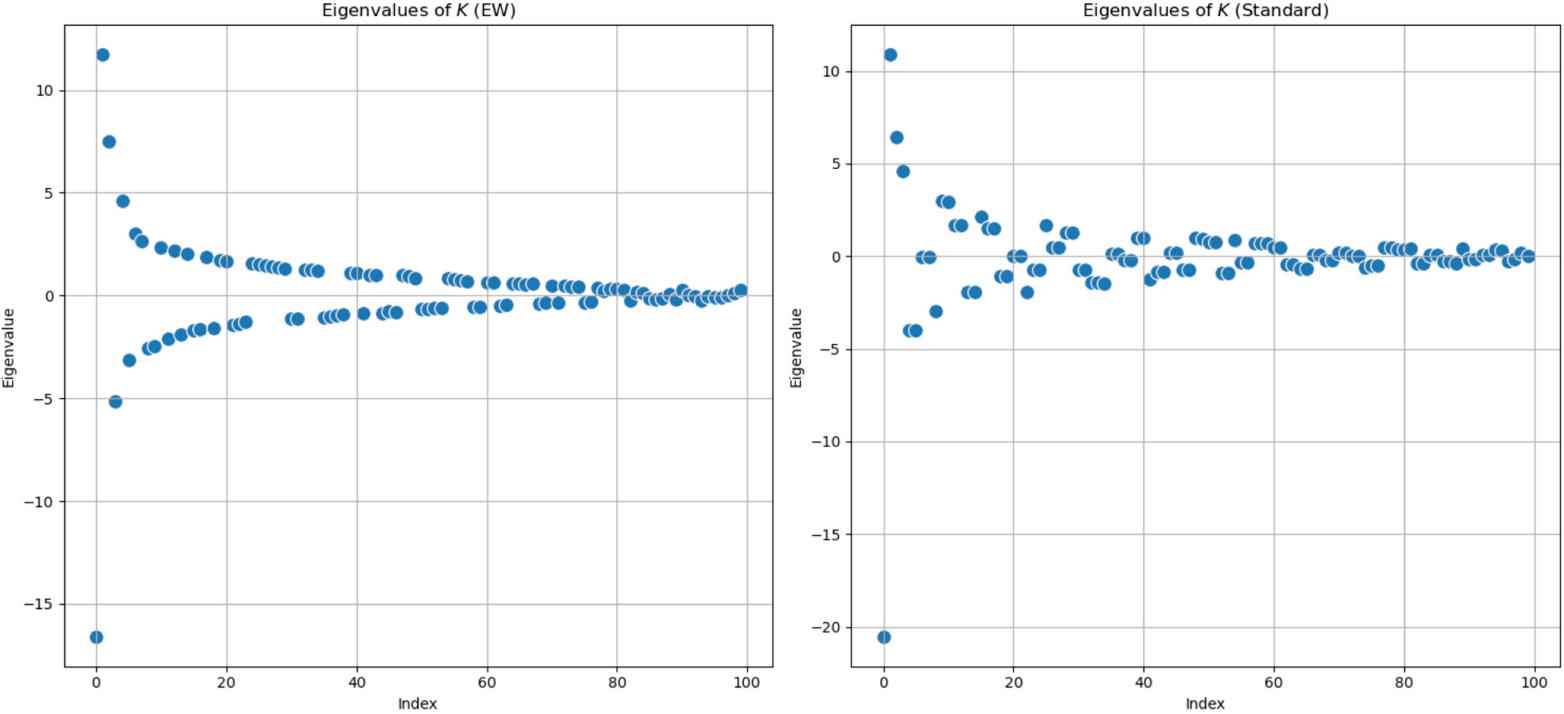
Eigenvalues of the *K*-operator plotted on the real plane. Left: eigenvalues of the *K*-operator computed with the element-wise product (the first product, the mixed one). Right: eigenvalues of the *K*-operator computed with the standard matrix product. In both cases, the inputted brain matrices $\mathcal {G}^k$ and $\mathcal {G}^{-1}$ are symmetrical. The *K*-operator is obtained by applying the $\mathcal {G}^k$ matrix of the diseased brain to the inverse of the matrix representing the healthy brain $\mathcal {G}^{-1}$

We show in Fig. 9 the results for the *K*-operator computed through the two kinds of product. We notice an oscillating trend, with the eigenvalues converging towards zero. We can see that for the element-wise multiplication, we have a symmetrical *K*-operator, thus all the properties deriving from this. With the element-wise matrix multiplication, the eigenvalues are perfectly distributed, see Fig. 9. In this case, the eigenvalues are very precisely distributed. This structure suggests a high level of order and regularity in the system. The precise distribution implies that the underlying connectivity retains strong symmetrical properties even after the action of the *K*-operator, so it indicates stability and regularity, typical of symmetrical systems. In the context of brain connectivity, this could reflect a highly ordered or balanced state. The distribution for the two methods of computation seems to be qualitatively similar, even if the point-wise product distributes the spectral changes in a more uniform way.

The same comparison has been conducted for new sparse matrices, as well. We consider entries of $\mathcal {G}^k$ that are different than 0 and are taken between 0 and 1. Both matrices $\mathcal {G}^k$ and $\mathcal {G}$ are symmetric, with sparsity characterised by non-zero entries randomly distributed between 0 and 1, and a small perturbation parameter $\sigma \ll 1$.^3^ This value of $\sigma$ denotes a high-degree of sparsity of the matrices, with non-zero entries scattered in a uniform way throughout the matrix itself. Refraining from introducing a structure within matrices in these tests, we could focus on properties of the product itself. In addition, we chose small matrices as as the most simple case. In the following, $100\times 100$-matrices will be considered as a progressive transition towards real-data matrices with a comparable number of rows and columns. As a general remark, even if the starting point is constituted by sparse matrices, their (matrix) product is less sparse, thanks to the non-zero element accumulation via multiplication. Changing the typology of the product also results in changes to the structure. This implies that a similarity of the structure of the output of two different kinds of products can shed light on some hidden similarities of the products themselves. Thus, the use of sparse matrices constitutes a good starting point for an empirical analysis of product features.

**Fig. 10:**
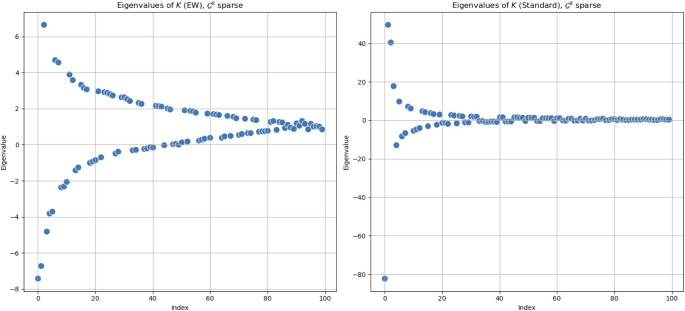
Eigenvalues of the *K*-operator, plotted on the real plane, obtained by the product of a symmetrical $\mathcal {G}^k$ with a symmetrical $\mathcal {G}^{-1}$, which are sparse matrices. Left: eigenvalues of the operator obtained with the element-wise, mixed product. Right: eigenvalues of the *K*-operator obtained with the standard matrix product

We present the results for the eigenvalues of the *K*-operators in Fig. 10, where we can see a deeper similarity between the two trends for the two different methods.

One more test has been implemented with a *K*-operator whose elements are the quotient of the corresponding elements of $\mathcal {G}^k$ divided by the corresponding elements of $\mathcal {G}$, choosing default small values to replace eventual 0 values in the $\mathcal {G}$ matrix:

18 $$\begin{aligned} {\left\{ K(t)\right\} _{ij} = \frac{\left\{ \mathcal {G}^k(t+1)\right\} _{ij}}{\left\{ \mathcal {G}^k(t)\right\} _{ij}}, } \end{aligned}$$

where *i*, *j* denotes two regions of interest, and $\left\{ K(t)\right\} _{ij}$ is the (*ij*)-th element of *K*(*t*). Thus, we compute the *K*-operator as the quotient of $\mathcal {G}^k$ divided element-wise by $\mathcal {G}$ with larger matrices. With this approach, we also avoid computing the inverse matrix before performing the product. This formula has been first used to investigate a seizure spreading from electro-corticography (ECoG) data from an epileptic patient [4]; here it will be applied for the first time to fMRI data, along with the eigenvalue analysis.

**Fig. 11:**
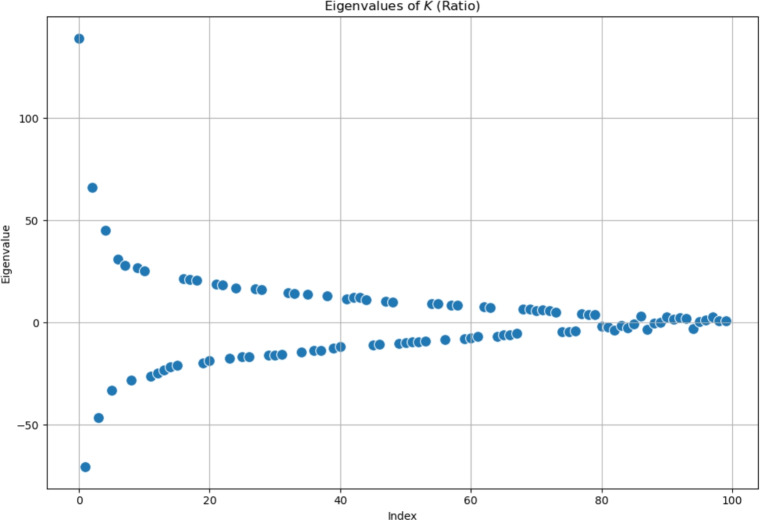
Eigenvalues of the *K*-operator obtained as element-wise ratio between $\mathcal {G}^k$ and $\mathcal {G}$, plotted on the real plane. $\mathcal {G}$ and $\mathcal {G}^k$ are symmetrical

Such a *K*-operator would act element-wise in an exact way, also being easier to interpret and justify. The results for the eigenvalue trend of this case can be seen in Fig. 11; comparing them with the previous results (Fig. 10), we notice an important correspondence between them and the standard product applied to sparse matrices.

We also compared these results obtained with simulated data with those obtained from real-life ones. In particular, we analysed the results concerning some real data from an Alzheimer-Perusini’s disease patient. The data are obtained from fMRI-derived DICOM files according to the procedure described in “A First Comparison of Eigenvalues for Real Data” section, from the ADNI dataset. We used the data of the first and second follow-ups of this patient to compute the *K*-operator through the aforementioned third product, Eq. 18, the ratio that leads to an exact form of element-wise product. We used the second follow-up as $\mathcal {G}^k$, the diseased brain, whether the first follow-up is our $\mathcal {G}$ healthy brain.

The last trial using larger symmetrical matrices was conducted with a particular operation, namely the so-called *kernel product*. This kind of product between two matrices is a matrix-product operation that is often used for the comparison of images. Given an element of a matrix, it works by multiplying the surrounding entries of the matrix; it employs a kernel function to measure similarities or relationships between rows (or elements) of two matrices. It is often used to define kernel methods in machine learning and computational mathematics.^4^

**Fig. 12:**
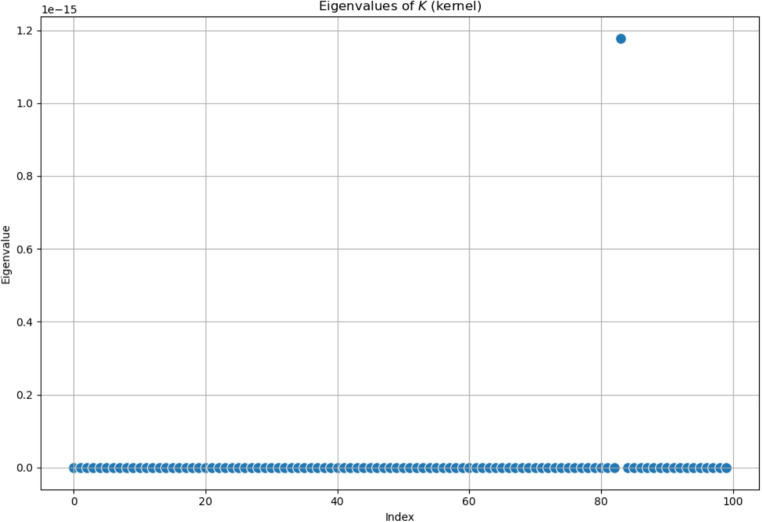
Eigenvalues of the *K*-operator obtained as kernel product between a symmetrical $\mathcal {G}^k$ and a symmetrical $\mathcal {G}$, plotted on the real plane. The Gaussian kernel was used

**Fig. 13:**
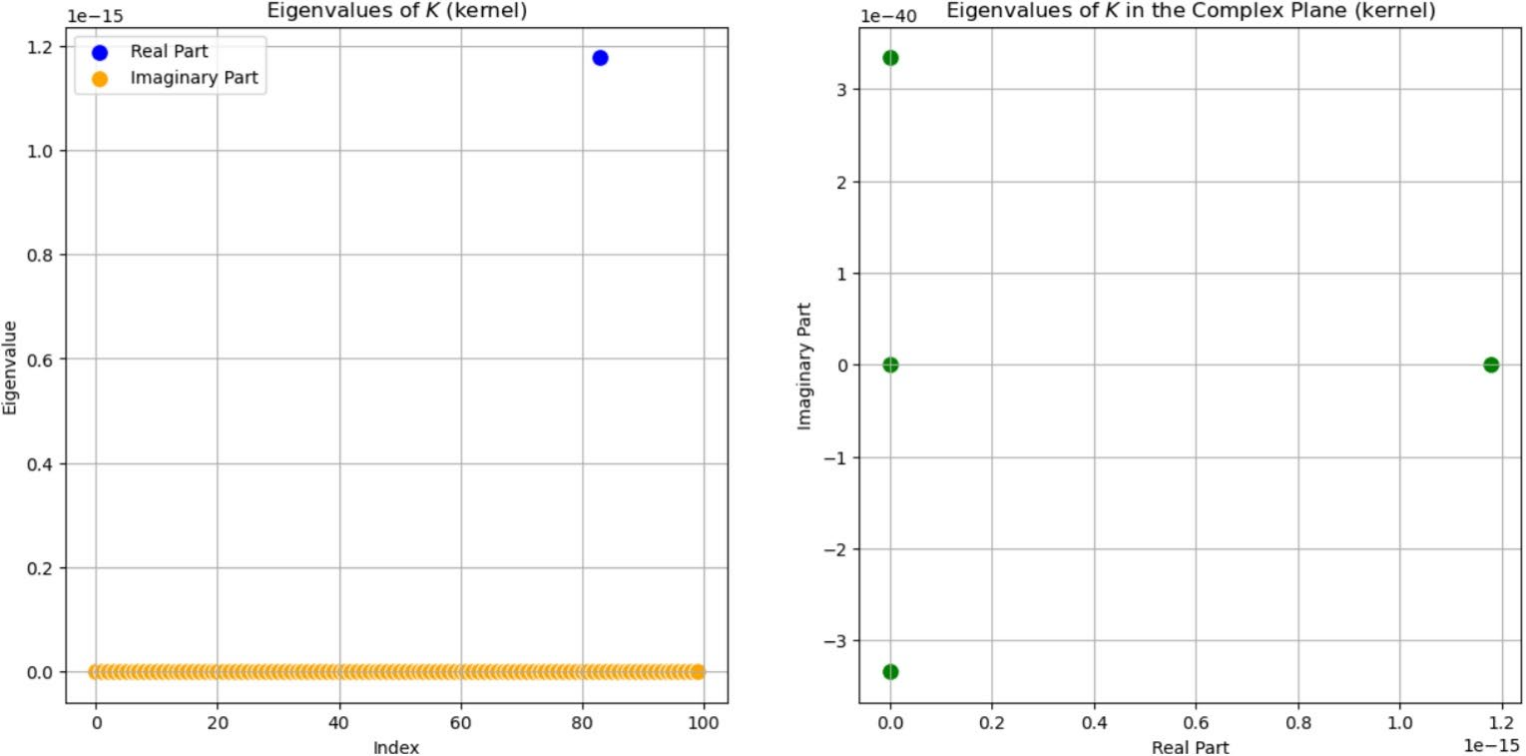
Eigenvalues of the *K*-operator obtained as kernel product between a symmetrical $\mathcal {G}^k$ and a symmetrical $\mathcal {G}$, plotted on the complex plane. The Gaussian kernel was used

The kernel product between two matrices does not lead to a symmetric result, unless the matrices are identical and the kernel function is symmetric itself. The obtained results, concerning the spectrum of the *K*-operator, are shown in Fig. 12, but we notice that the results show evident differences when compared with the previous ones. In fact, all eigenvalues are set to a null value, except for the only one that stands out; surprisingly, it is not the first one, as we are used to. We also tried to show the results in the complex plane, in Fig. 13. Maybe these exceptions could lead to a more general property, but we postpone this subject to future studies.

## Using *K* to Detect Dynamics in Simulated Data

Let us now explore the *K*-operator’s properties from a dynamical point of view. We aim to see if we can study and predict the time evolution of this mathematical object. First, we will work with simulated data, in particular, we will investigate three specific simulations to analyse the time evolution of $\mathcal {G}(t)$ and *K*(*t*), assessing how an initially healthy brain is progressively damaged by the onset of a disease. Later, we will use real data to investigate how the disease-provoked brain damage progresses over time.

We will compare the time evolution of $\mathcal {G}^k(t)$, generating a null model, where all matrix entries are completely random yet bounded between 0 and 1. Then, a model where the values are increasing, one with decreasing values, and finally, one with varying values, where all entries first increase and then decrease. We will compare a model that has no constraints and three different models that have one. The four models have a similar base set-up; we generate an arbitrary number of matrices, specifically 10, which are representative of the time evolution of a diseased brain. These matrices have a size of $4\times 4$ and have randomly-generated entries, whose values are bounded between 0 and 1. For what concerns the other three models, the only difference is that in the increasing one, the values of the entries are increased step-by-step by an arbitrary values randomly chosen between 0.01 and 0.05; in the decreasing set-up, we decrease the all the entries’ values by the same quantity, while in the varying model, the values are increased up to the middle point, then they are progressively decreased.

Successively, for every model, we perform a linear regression on the matrices to find the discrete $\mathcal {G}(t)$, labelling each time point, and thus each matrix, with natural numbers from 1 to 10. After having obtained the coefficients from the linear regression for each matrix element independently, we compute the continuous $\mathcal {G}(t)$ through the linear model for a specific continuous time, using Eq. 20.

20 $$\begin{aligned} G^k(t) = at + b. \end{aligned}$$

Nevertheless, the linear regression does not describe the time evolution of $\mathcal {G}(t)$ well, which is why we also computed its quadratic regression. The equation used to represent the continuous form of $\mathcal {G}(t)$ with a quadratic regression is Eq. 21:

21 $$\begin{aligned} G^k(t) = a t^2 + b t + c. \end{aligned}$$

**Fig. 14:**
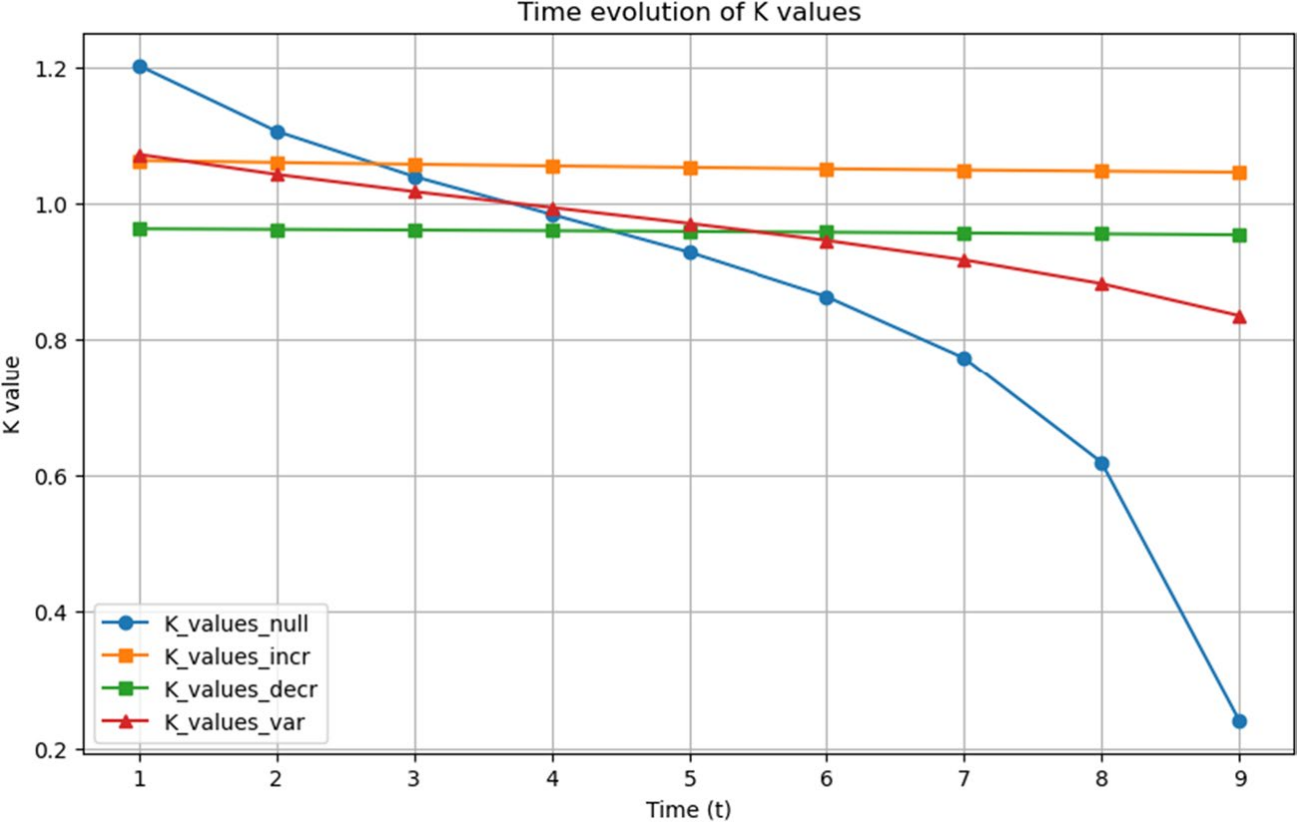
Comparison of the four different models where all four trends of the first single value for the *K*-operator are shown

Once the $\mathcal {G}(t)$ evolution of the progressively-diseased brain has been analysed, we subsequently compute from it the time-evolving *K*-operator. The operator is computed as the ratio of the successive $\mathcal {G}(t)$ matrices, using the method we had already explored in “Extending the Analysis to Larger Matrices, and Adding a Third Definition of *K*” section, as an exact element-wise product, see Eq. 18. We show in Fig. 14 a comparison for a single element of the *K*-operator matrix, obtained for the four different models. The shown element was arbitrarily decided to be the first entry.

If the behaviour of the single *K*-operator element was representative of the global behaviour of *K*, we could infer that the operator detects the dynamics of the system. In fact, as we can see from Fig. 14, the *K*-operator successfully catches the differences in the models, because there are evident differences in the action range of the operator. For what concerns the increasing model, the range is stabilised above 1, denoting a factor that increases the entries of the brain matrix, while for the decreasing model, we have a value above 0 but below 1, confirming the decreasing action on the brain matrix. Finally, for the variable model, that is, an increasing-decreasing model, we have a segment above 1 and another one below it, still above 0. The null model yields a completely different behaviour.

## An AD Case Study, Focusing on a Pair of ROIs

Now we want to use the *K*-operator to focus on some pairs of ROIs and begin to think about a possible analysis of the dynamics of *K* for those sub-matrices. We will also show another kind of analysis, namely the recurrence plots RPs. In particular, we inspect the time evolution of a diseased brain $\mathcal {G}^k(t)$, using real data obtained from a female patient affected by the Alzheimer-Perusini’s disease. Specifically, the data used are fMRI data from the dataset ADNI, from a patient labelled as $019\_S\_5019$. A part of the results of this section has also been reported in the first author’s MSc thesis [19].

**Fig. 15:**
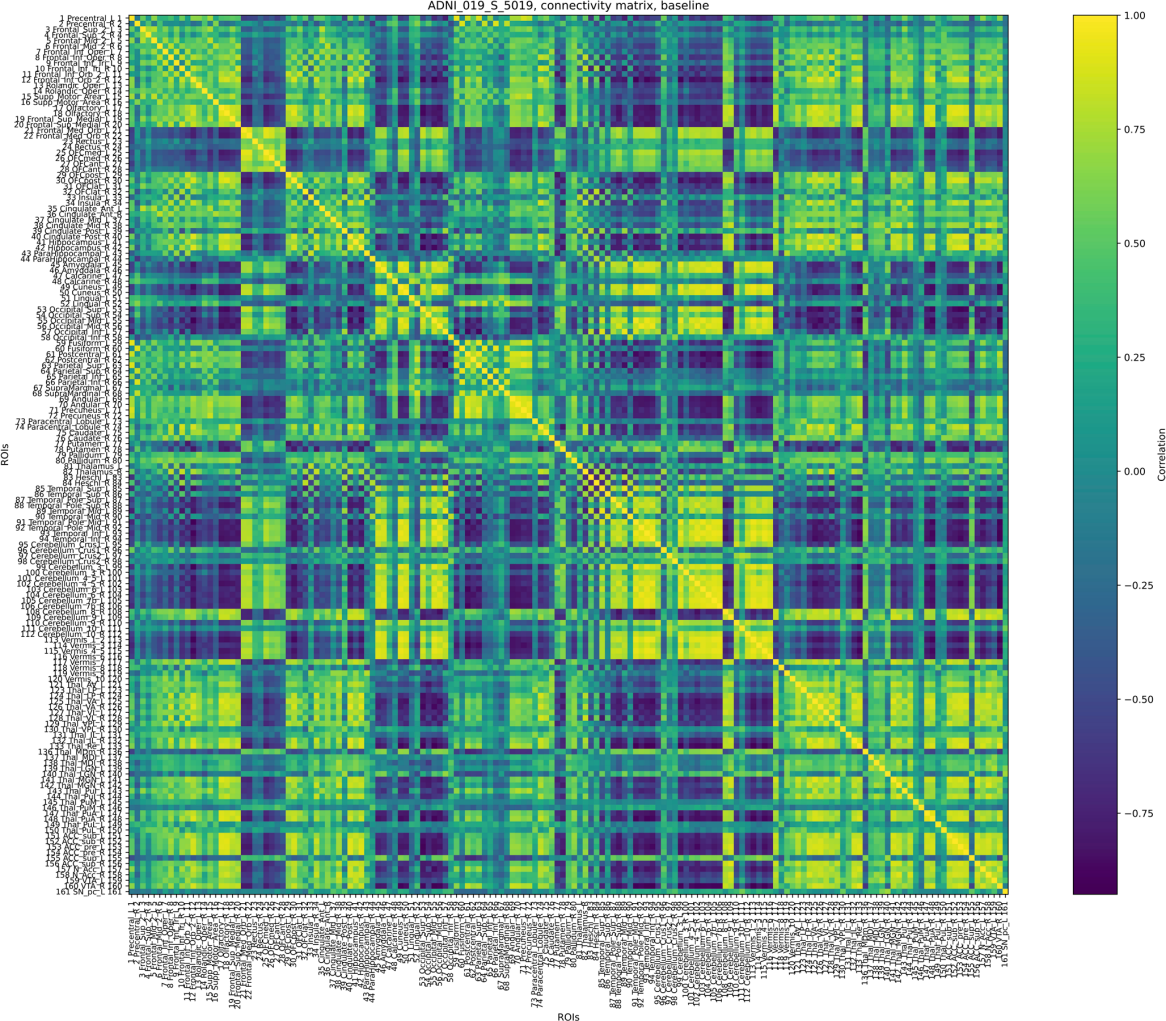
Connectivity matrix of a female AD patient at the baseline, $\mathcal {G}^k_0=\mathcal {G}^k(t_0)$

**Fig. 16:**
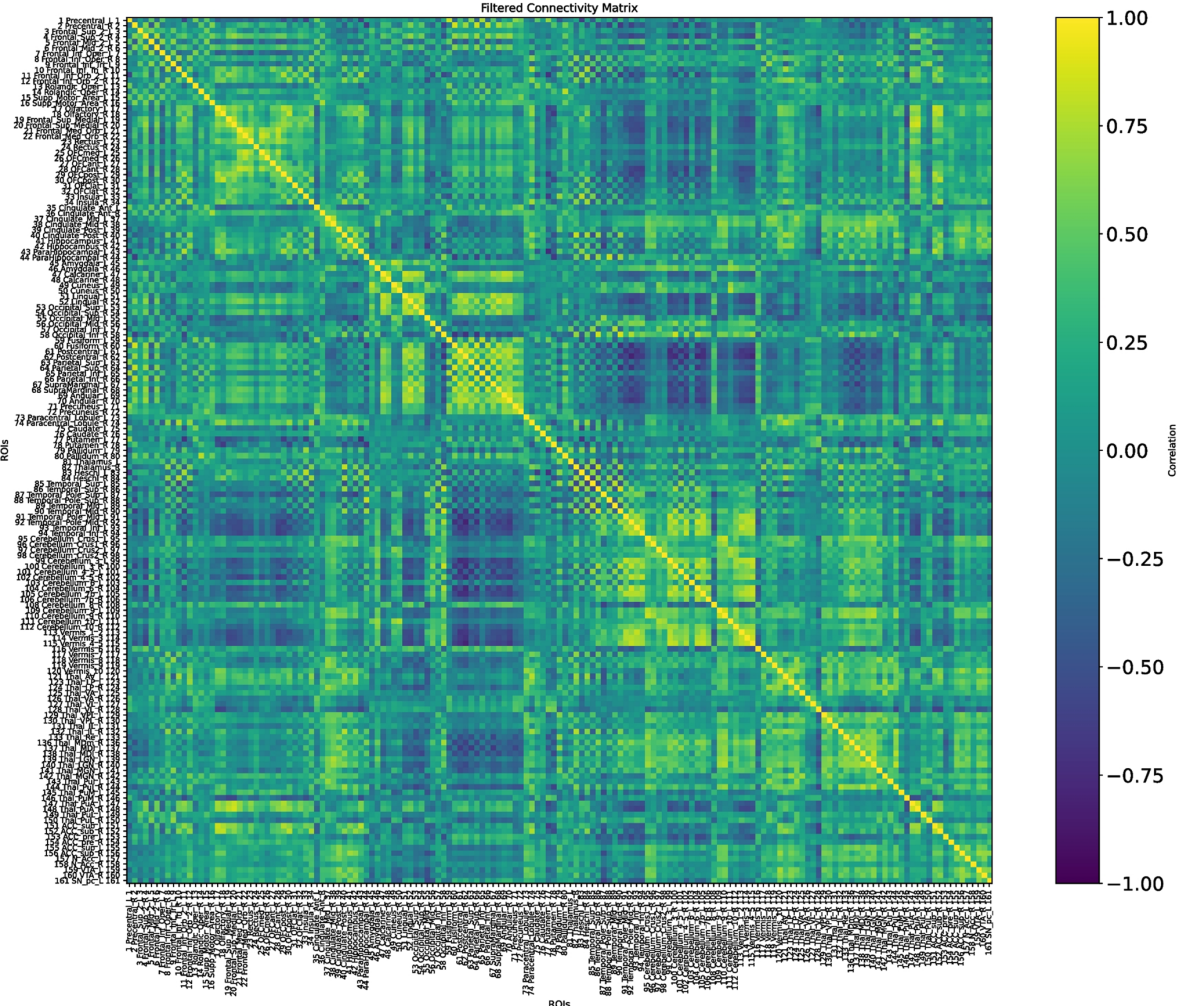
Connectivity matrix of the same AD female patient at the first follow-up, $\mathcal {G}^k_1=\mathcal {G}^k(t_1)$

**Fig. 17:**
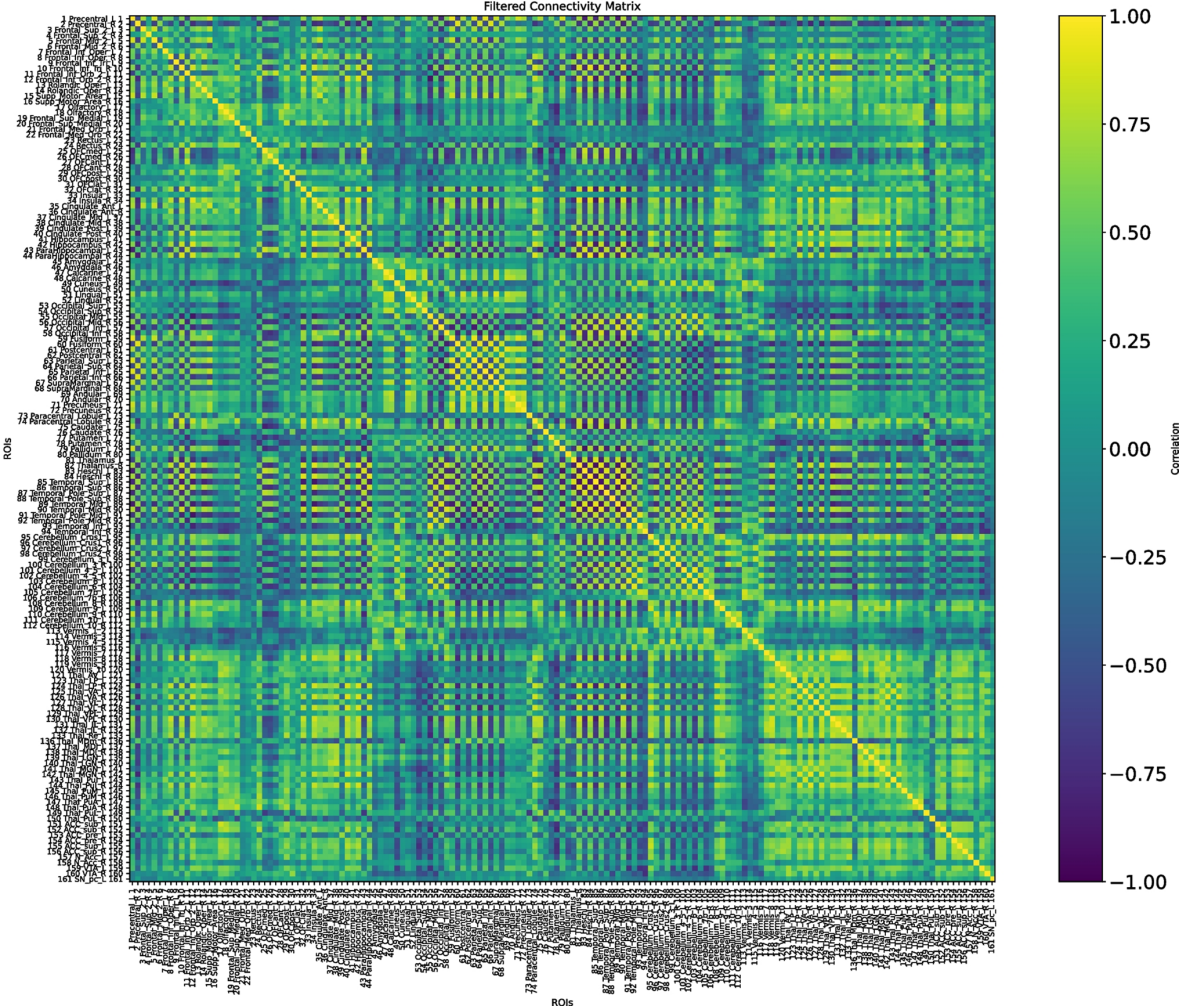
Connectivity matrix of the same AD patient at the second follow-up, $\mathcal {G}^k_2=\mathcal {G}^k(t_2)$

The fMRI data were taken at three different moments of the disease: at the baseline, when the disease is still not too extended; at the first and second follow-ups, when we can assume that the disease is already at an advanced stage. The distance between the baseline and the first follow-up is four months, while three months separate the first and second follow-ups. Figures 15, 16, and 17 show $\mathcal {G}^k_0,\,\mathcal {G}^k_1,\,\mathcal {G}^k_2$, respectively.

We computed a connectivity matrix for each one of the stages, considering them as $\mathcal {G}^k(t_0)$ (baseline), $\mathcal {G}^k(t_1)$ (first follow-up), and $\mathcal {G}^k(t_2)$ (second one follow-up).

Before delving into the detailed observation of the *K*-operator, we propose here a computation of the symmetry and modularity of the $\mathcal {G}^k$ matrices and of *K*. Since the connectivity matrices are symmetric (undirected connectome) and they do not contain null elements, their sparsity is 0, and their asymmetry is 0 as well. Imposing a threshold, and thus forcing the entries below to be null, we can retrieve some information on the sparsity and thus on the structure of the matrix. We can also compute the modularity of the brain matrices, directly from the connectome, and the sparsity of the *K*-operator, see Table 1, obtained according to the definitions (modularity, from [20]):

22 $$\begin{aligned} {\begin{matrix} & \text {sparsity}(A) = 1 - \frac{\left| \{ (i,j) \mid A_{ij} \ne 0 \} \right| }{n \cdot m}\\ & \text {modularity}(A) = \frac{1}{2m} \sum _{i,j} \left[ A_{ij} - \frac{k_i k_j}{2m} \right] \delta (c_i, c_j), \end{matrix}} \end{aligned}$$

where:

- $A \in \mathbb {R}^{n \times m}$ is the considered matrix;
- $\Vert \cdot \Vert _F$ is the Frobenius norm;
- $A^\top$ is the transpose of $A$;
- $n, m$ are the matrix dimensions;
- $m = \frac{1}{2} \sum _{i,j} A_{ij}$ is the total edge weight;
- $k_i = \sum _j A_{ij}$ is the degree of node $i$;
- $c_i$ is the community assignment of node $i$, and
- $$\delta (c_i, c_j) = 1$$ if $$c_i = c_j$$, else 0.

Our results are presented in Table 1. The computation of modularity required an overall shift of the values, to get all positive numbers: +1 for $\mathcal {G}s$, +1000 for *K*. We notice the dramatic increase in sparsity from the baseline to the first follow-up, less evident between the latter and the second follow-up. The information is mirrored by the *K*-operator, whose overall action is stronger between the first two time points (sparsity of $K_{01}$ equal to 0.28), than between the second and the third one (sparsity of $K_{12}$ equal to 0.17). The modularity of the *K*s is comparable. The information is provided by the different, local numerical values; some of them will be considered in the following to choose the brain regions to focus on. The progressive reduction of modularity of the connectivity matrices is also evident. However, this information cannot be directly compared with the modularity of $K_s$, given the different numerical values and the point-wise nature of the third product. For the sake of completeness, we also included a comparison with the sparsity and modularity of the *K*s computed with the other two products. For both the mixed product $K^{*}$^5^ and the standard product $K^{@}$^6^, there is a decrease in modularity, more dramatic for the standard product. And, also for $K^{*}$ and $K^{@}$, the impact of the operator is higher between the baseline and the first follow-up.

**Table 1:** Values of measures for connectivity matrices and *K*-operators (computed with the products purely element-wise, mixed, and standard, respectively)

	$\mathcal {G}_0^k$	$\mathcal {G}_1^k$	$\mathcal {G}_2^k$	$K_{01}$	$K_{12}$	$K_{01}^{*}$	$K_{12}^{*}$	$K_{01}^@$	$K_{12}^@$
Sparsity	0.35	0.77	0.41	0.28	0.17	0.8124	0.4706	0.0363	0.0200
Modularity	0.06	0.04	0.10	0.00338	0.00328	0.0040	0.0039	0.06894	0.00394

For the sparsity, a threshold of 0.3 has been applied

As a general caveat, we cannot directly compare the *K*-operator against established measuring techniques, because we aim to propose and develop a more theory-based, general overview of disease that can include specific cases. The *K*-operator can be used in preliminary steps of analysis, as will be shown in the following, where we can select brain areas according to some values of *K*, and then perform an analysis with established techniques. We also want to stress that the *K*-operator is in principle a time-evolution operator. It describes how something (the functional connectome in our current definition) changes, not how something is defined.

**Fig. 18:**
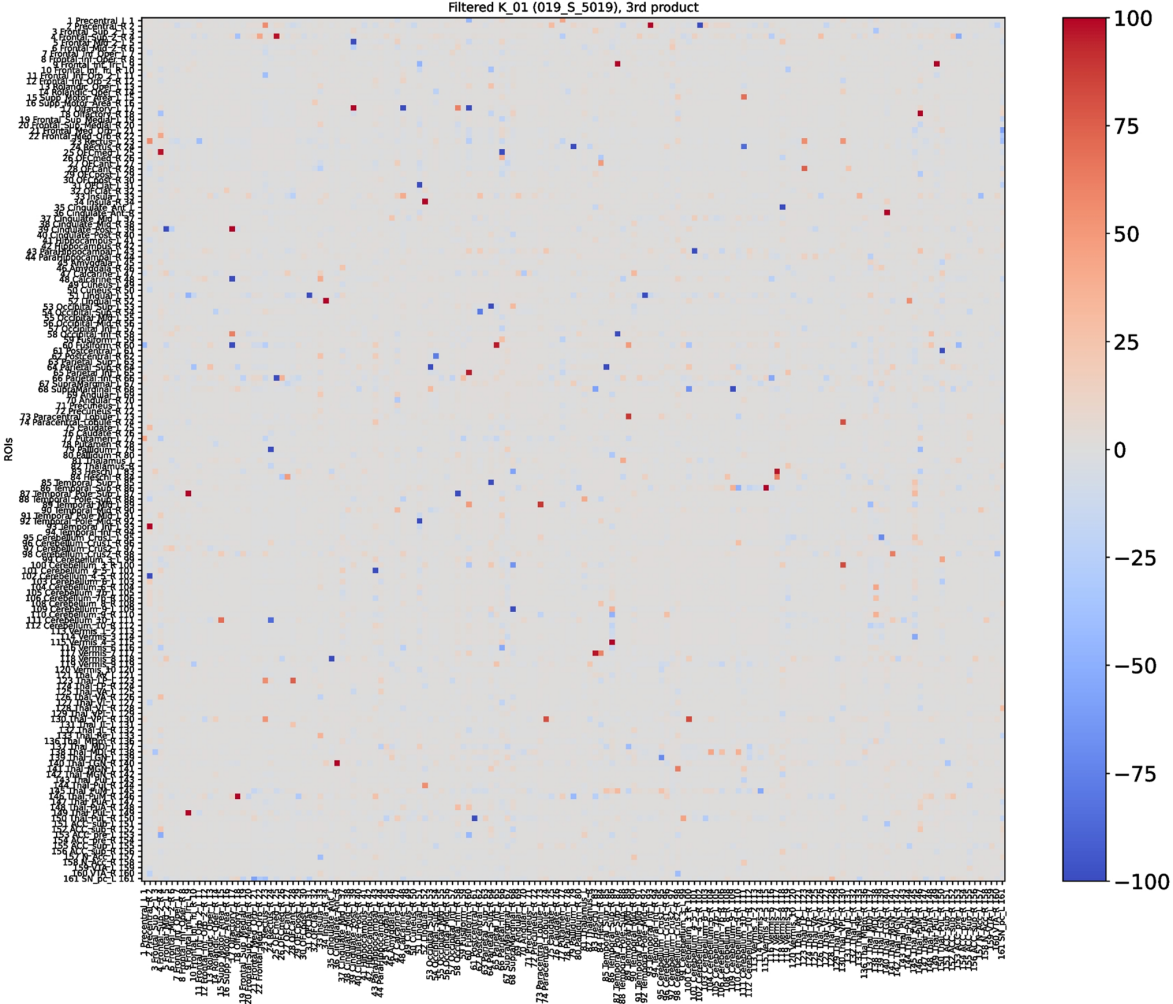
*K*-operator for the considered AD patient, between the baseline and the first follow-up

**Fig. 19:**
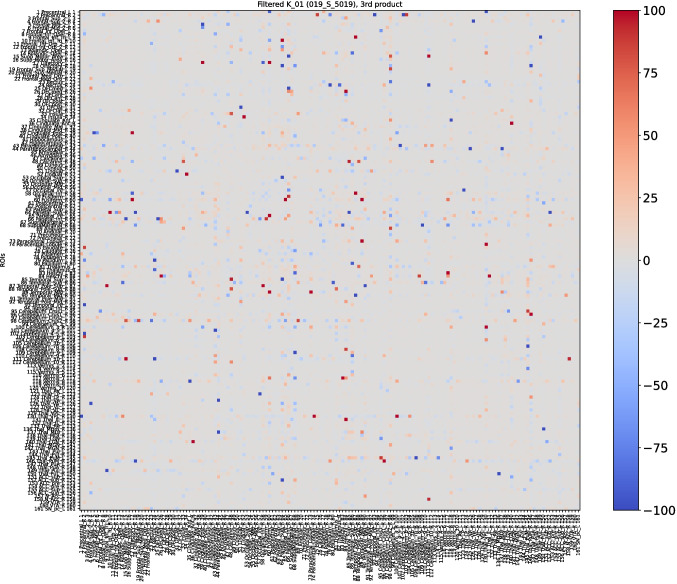
*K*-operator for the female AD patient, between the first and the second follow-up

**Fig. 20:**
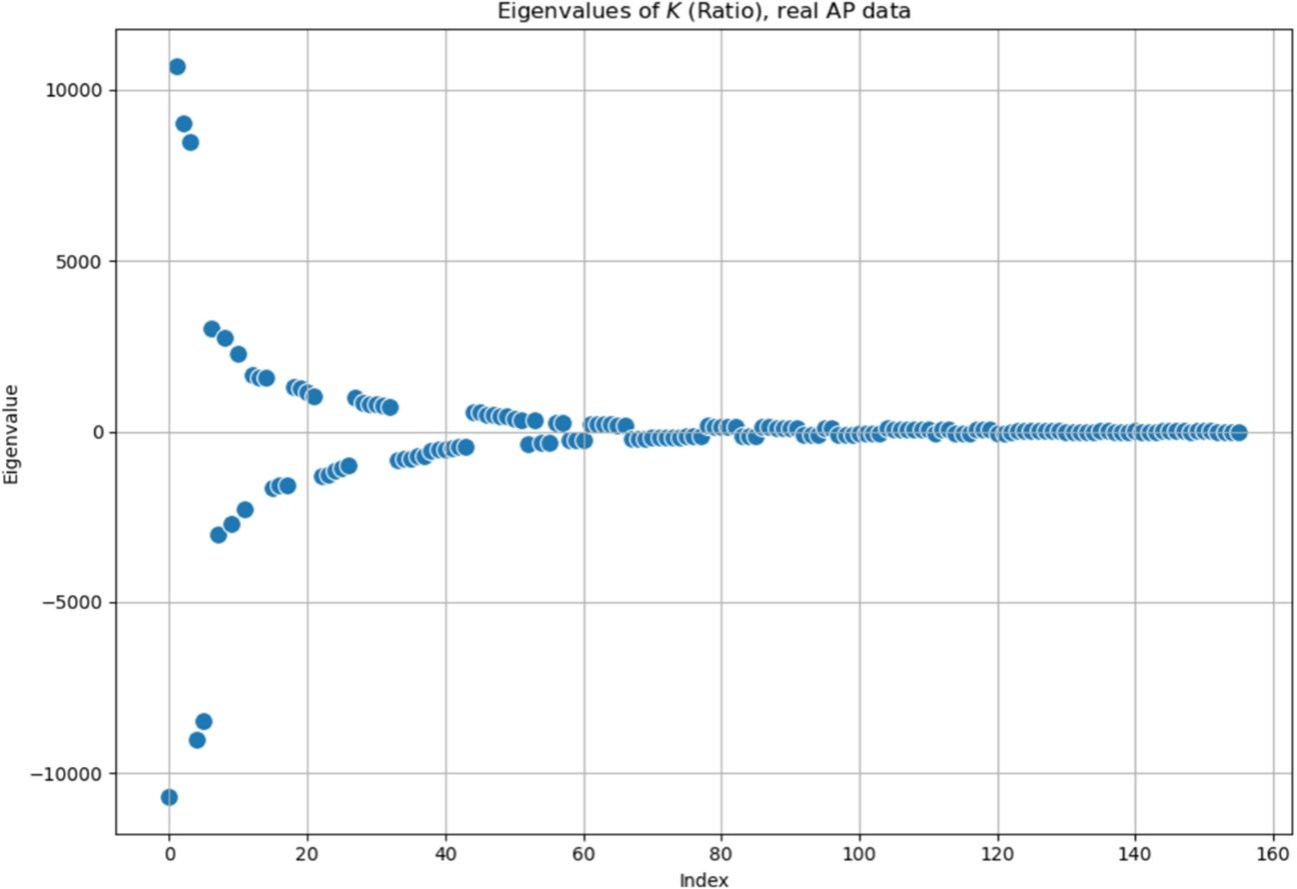
Eigenvalues of the *K*-operator obtained from real data as third product, the ratio between $\mathcal {G}^k(t+1)$, the second follow-up, and $\mathcal {G}^k(t)$, the first follow-up

Baseline and first follow-up of this patient were already examined with the first and second definition of the *K*-operator in [12]; here, we do also include the second follow-up, and we perform the analysis with the third definition of *K*, with the *ratio*. However, rather than focusing on the global information of the brain connectome, we will choose a specific pair of regions. As a first step of the analysis, we show here the *K*-operator computed between the baseline and the first follow-up, and between the first and the second follow-up, computed via the purely element-wise product (the third type of product in our analyses), see Figs. 18 and 19. The chosen atlas is AAL3 [21]. Before moving forwards with the analysis of some submatrices of *K* and with a focus on specific brain regions, we present the patterns of eigenvalues from $K_{01}$ (Fig. 19), shown in Fig. 20. We notice a decreasing trend from both sides of the *x*-axis, where we can see once again that the eigenvalues assemble in groups.

Observing the values of the *K*-operators $K_{01}$, between baseline and first follow-up (Fig. 18), and $K_{12}$, between the first and the second follow-up (Fig. 19), we choose to focus on two specific pairs of ROIs that denote particular changes over time, also taking into account their importance for disease progression. Among the elements of *K* showing higher absolute values, the ones on which we focused are the regions of interest 112 and 159, corresponding, respectively, to the Cerebellum_10_R and the VTA_L (ventral tegmental area, left).

We selected this pair of ROIs for the high value of the corresponding element of the *K*-operator, and for their interest in the analysis of the disease. VTA is part of the dopaminergic system, and its alteration may lead to hippocampal dopaminergic alterations, related to memory deficits [22], and functional connectivity of VTA constitutes one of the early markers of AD onset [23]. A high value of the *K*-operator between the first and the second follow-up in this area could signal a compensatory mechanism. An in-depth analysis of cerebellum 10 R and VTA can be the object of more research in itself.

**Fig. 21:**
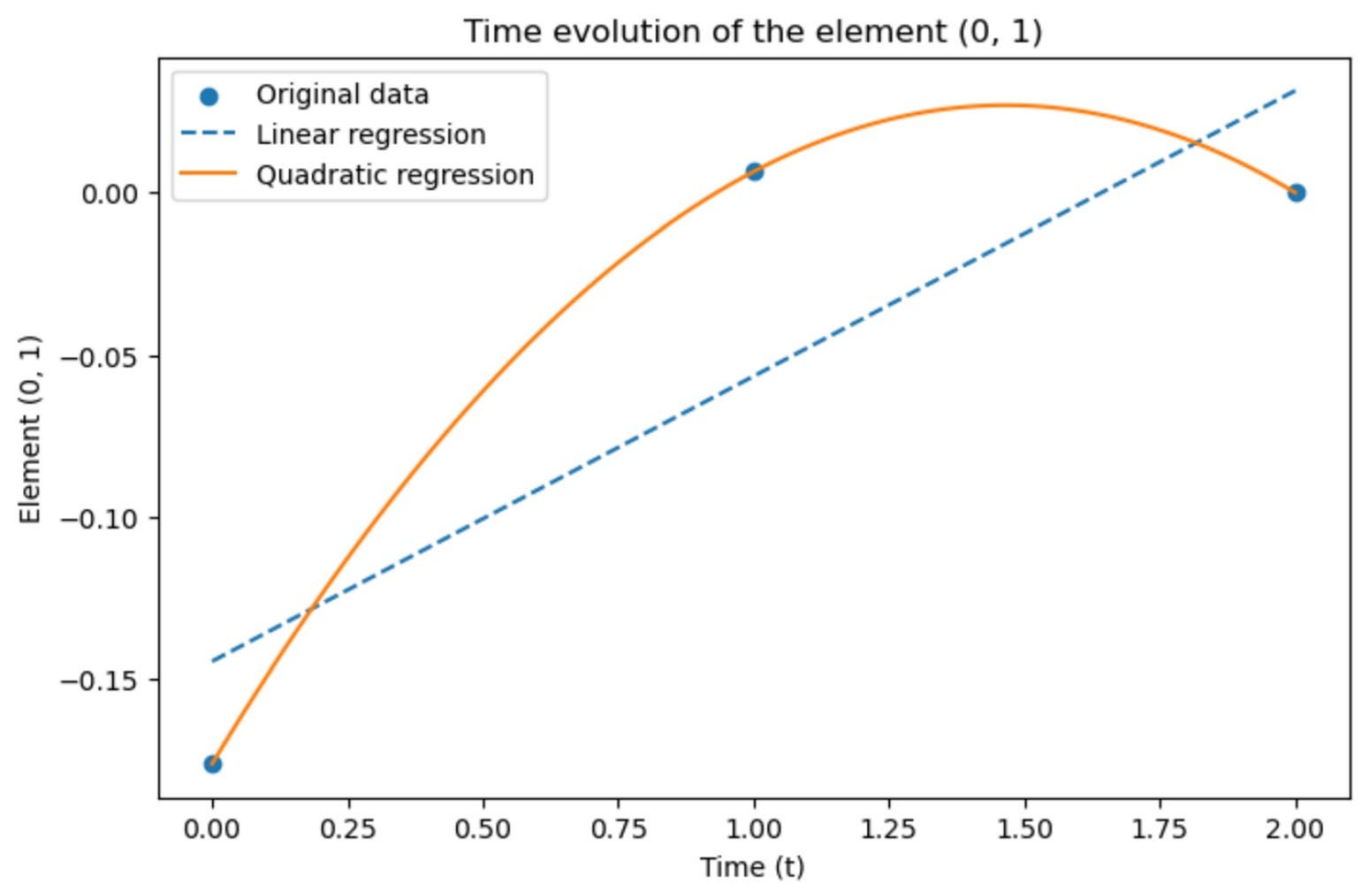
Time evolution of the element [0, 1] of $\mathcal {G}^k|s(t)$ from real data

**Fig. 22:**
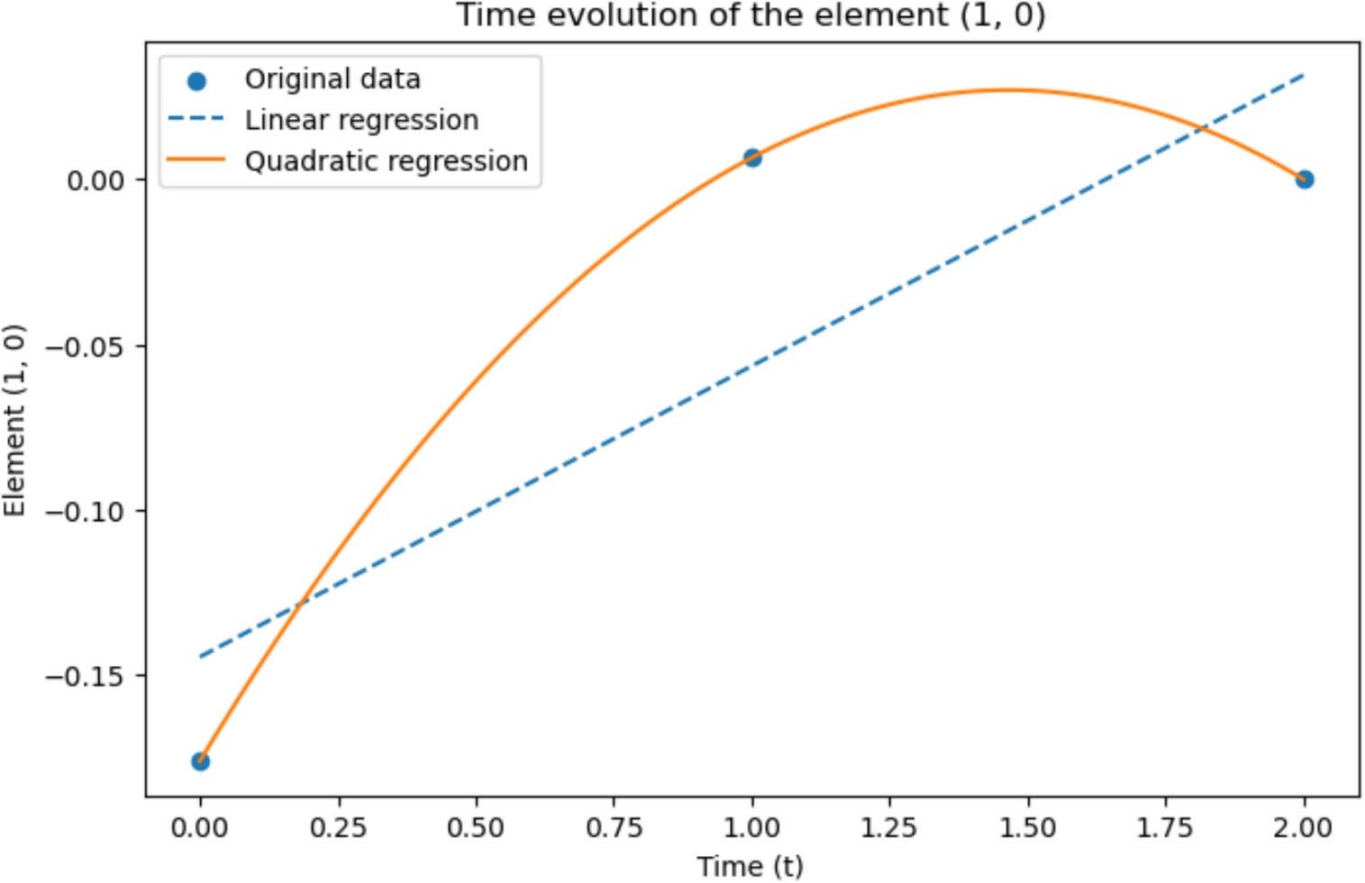
Time evolution of the element [1, 0] of $\mathcal {G}^k|s(t)$ from real data

To focus our analysis on these regions, we considered again the $\mathcal {G}^k(t_0),\,\mathcal {G}^k(t_1),\,\mathcal {G}^k(t_2)$ matrices, and from each of them, we extracted a submatrix of the size of $2\times 2$, analysing the time evolution of each of its elements, considered independently. We indicate the selected submatrix as $\mathcal {G}^k|_s(t_i)$, where *s* stands for “submatrix” and $t=0,1,2$, and its elements are denoted as $\mathcal {G}^k|s(t_i)[i,j]$, with $i,j = 0,1$. Thus, our analysis focused on the interaction between ROIs 111 and 158 from the correlation matrices of baseline, first and second follow-ups, considering now these sub-matrices as our new $\mathcal {G}^k(t)$, that is more precisely indicated as $\mathcal {G}^k|_s(t)$, at the three different time points. Successively, we analysed the time evolution of every single element of the submatrices through a linear regression and a quadratic one. The results are shown in Figs. 21 and 22, respectively.

Concerning the two diagonal elements of the submatrices, the related figures are missing because their values are constantly 1 in time: in fact, the initial connectivity matrices are uniform on the diagonal, given the perfect correlation with each region with itself. The other two elements that are shown in Figs. 21 and 22 have an interesting pattern, since their value, initially always negative, becomes positive in time, with a last decrease between the second and third time-step. It seems that, with the spreading of the disease, these regions of the brain tend to be more mutually active at first, but then their mutual correlation decreases. As currently defined, the $\mathcal {G}^k$ does not show information on the activity inside each brain area, but on the correlation between different brain areas. These assumptions are made from a small amount of data; this study should be extended for a more precise definition of both the theoretical approach and experimental results. In the same figures, we also included linear and quadratic regression. As expected, the quadratic regression shows a perfect correspondence with the precise values. However, only three data points have been considered, given the limited data availability, and thus, it is not possible to realistically measure the precision of the modelling.

The brain’s function is highly dynamic and can be viewed as a dynamical system. Therefore, we use recurrence analysis, specifically a recurrence plot (RP), to characterise its behaviour. An RP is a visualisation of a square matrix, whose entries represent the state in which the dynamical system recurs, that is, when the trajectory returns to a region in the phase space where it was before [24]. In particular, when using fMRI, as in our study, the characteristic non-stationarity of the data is taken into account through a recurrence analysis [25–27], which aims to compare and catch similarities between pairs of time series.

In fact, the signal of fMRI-derived time series is often nonlinear and non-stationary, and multivariate (multiple regions can be activated together, and thus they are observed simultaneously). Recurrence analysis can thus help characterise the behaviour of the time series, also providing a time-by-time comparison, and a comparison of dynamics across different ROIs. An RP is defined as:

23 $$\begin{aligned} R_{i,j} = \Theta (\varepsilon - \left\| \textbf{x}_i-\textbf{x}_j\right\| ), \end{aligned}$$

where $\Theta$ is the Heaviside function, $\varepsilon$ is the recurrence threshold, $\Vert \cdot \Vert$ is a norm, and $\textbf{x}\in \mathbb {R}^n$ is the state vector. In addition to RP, we also considered the joint RP [28], calculated as the Hadamard product of the RPs of two systems:

24 $$\begin{aligned} JR_{i,j}^{a,b} = R_{i,j}^a * R_{i,j}^b, \end{aligned}$$

where $\textbf{R}^a,\,\textbf{R}^b$ are the RPs of the first (*a*) and the second system (*b*), respectively. We then compute the recurrence rate (i.e., the probability of recurrence) (RR), which measures the density of points in the RP, defined as in Eq. 25 [24]:

25 $$\begin{aligned} RR = \frac{1}{N^2} \sum _{i,j=1}^{N} R_{i,j}. \end{aligned}$$

Similarly, we can calculate the RR from the joint RP, indicating the recurrences that occur simultaneously in both systems:

26 $$\begin{aligned} JRR = \frac{1}{N^2} \sum _{i,j=1}^{N} JR_{i,j}, \end{aligned}$$

ranging between 0 and *RR* of the individual RPs. Large *JRR* values ($JRR \approx RR$) indicate generalised synchronisation, where vanishing *JRR* values correspond to non-synchronous systems [24].

**Fig. 23:**
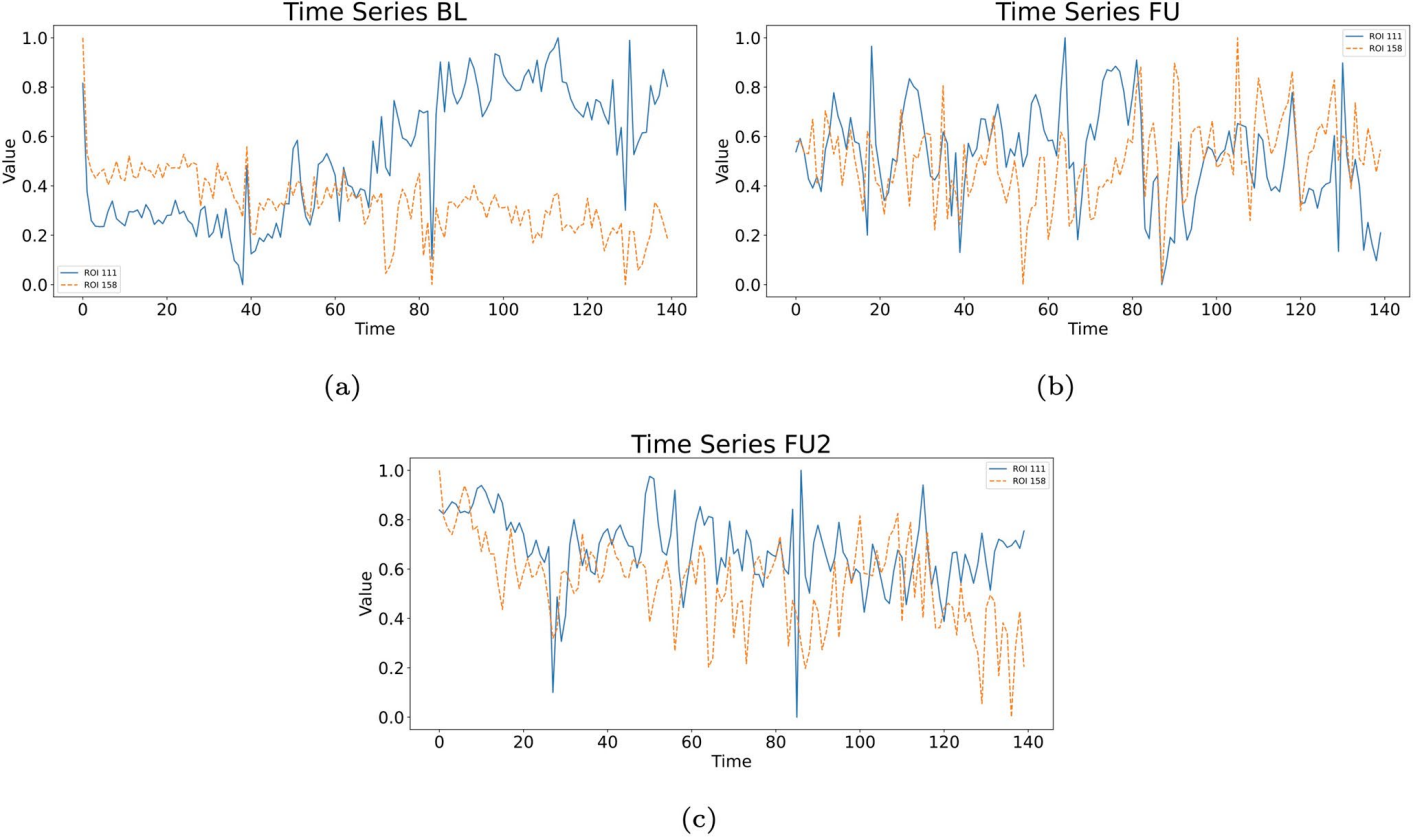
Time series of the fMRI signal for ROIs 112 (Python numbering 111) and 159 (Python numbering 158), at the baseline (a), first follow-up (b), and second follow-up (c)

**Fig. 24:**
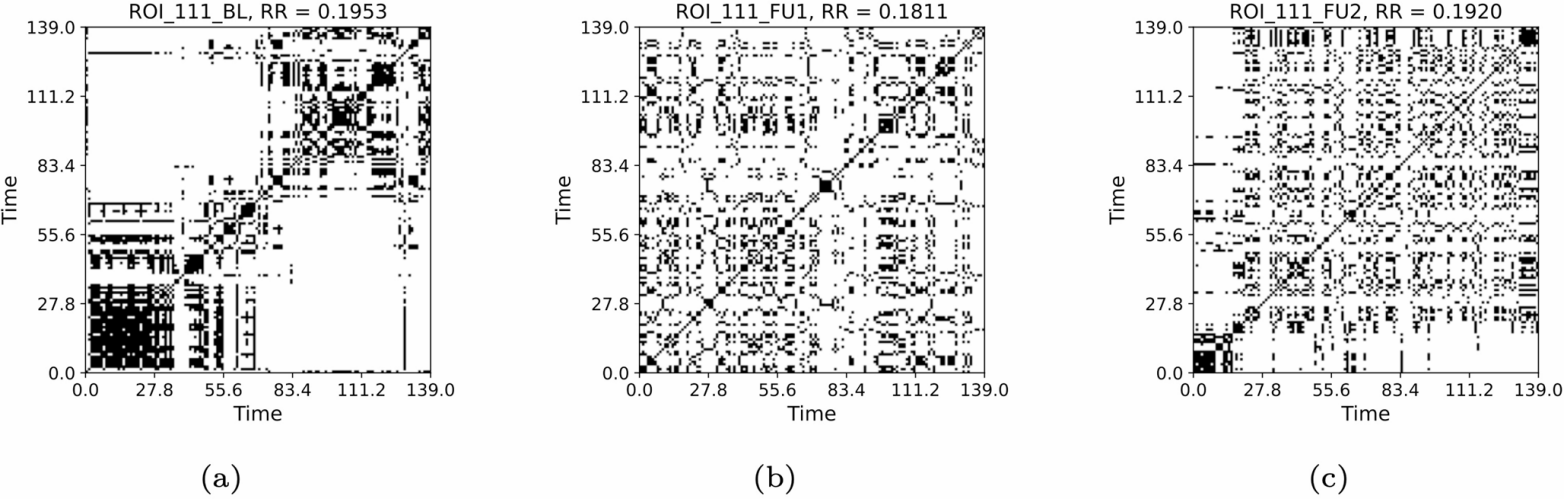
Recurrence plots for time series of ROI 112 (Python numbering 111), that is, Lobule X right of the cerebellum, at the baseline (a), first follow-up (b), and second follow-up (c). Recurrences ($R_{i,j} =1$) are indicated in black

**Fig. 25:**
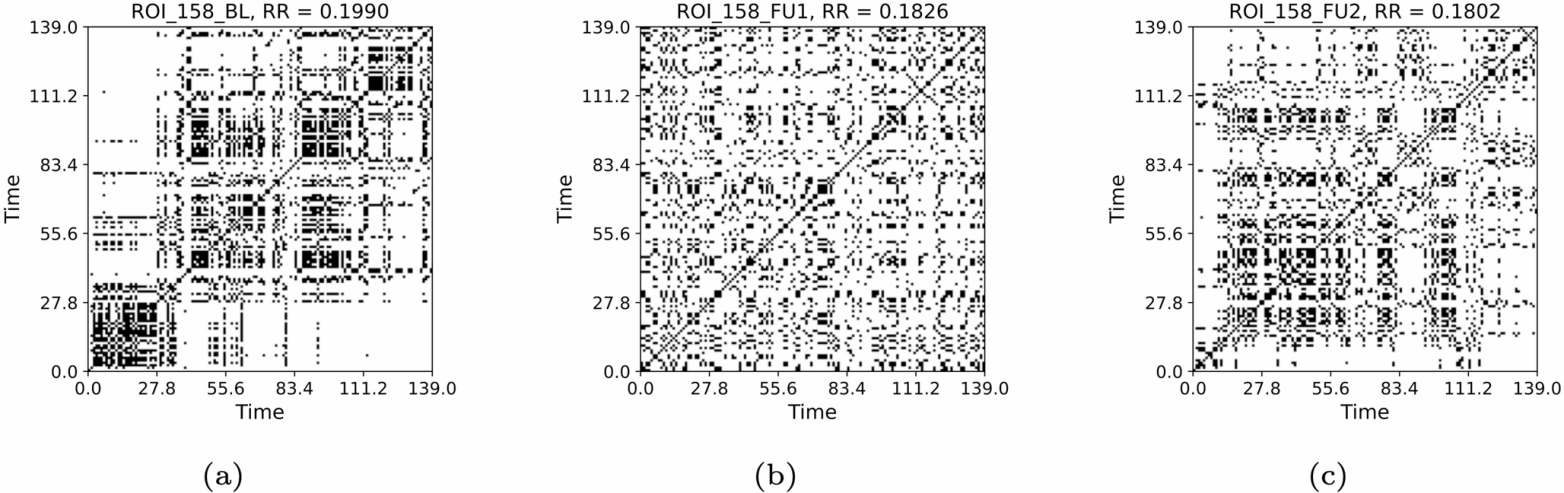
Recurrence plots for time series of ROI 159 (Python numbering 158), that is, VTA left, at the baseline (a), first (b), and second (c) follow-up. The recursions are indicated in black. Threshold 0.5

**Fig. 26:**
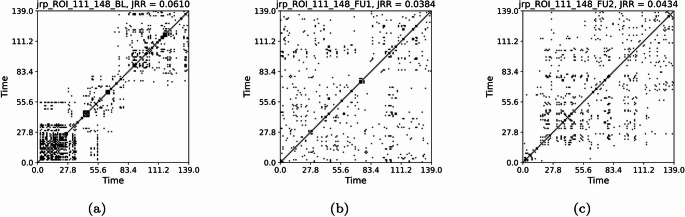
Joint recurrence plots for time series of ROI 112 and 159

From the time series of Lobule X right of cerebellum and VTA left (Fig. 23), we compute the RPs presented respectively in Figs. 24 and 25, and their joint RPs (Fig. 26), at the baseline ($t_0$), first follow-up ($t_1$), and second follow-up ($t_2$). We consider in all cases a threshold of $\varepsilon =0.3$ and normalised time series, and use the Euclidean norm (default for pyts.image.RecurrencePlot), whereas time-delay embedding is not used.

From the observation of the RPs, from $t_0$ to $t_1$ we notice a progression towards homogenisation, with the disruption of the patterns, for both regions. However, the passage to $t_2$ implies a partial re-organisation of the pattern. This could be associated with the re-organisation of behaviour occurring with the progression of the disease. The values of the RRs for the Lobule X present a decline between $t_0$ (0.1953) and $t_1$ (0.1811), but an increase for $t_2$ (0.1920), denoting a partial re-organisation (Fig. 24) However, the RR shows a slower but constant decrease for the VTA, with 0.1990 at $t_0$, 0.1826 at $t_1$, and 0.1802 at $t_2$ (Fig. 25). From the JRPs, it is visible a dramatic disruption of the coordination between the two regions (Fig. 26). Also the JRR shows a decrease from $t_0$ to $t_1$ (0.0610 to 0.0384), followed by an increase at $t_1$ (0.0434). Thus, we see a decrease in the values, that we could carefully interpret as a decrease of synchronisation with progression of the disease.

Re-organisation of the functional activity does often occur in neurological disorders. In particular, in our AD case study, we focused on the interaction between cerebellar lobule X (in particular, Cerebellum 10 R) and the ventral tegmental area (VTA), left. This is relatively unexplored in the literature, constituting a reason of interest. However, the regions of the vermis of the cerebellum have been more explored, also in relation to AD. Also, cerebellar atrophy and progression of atrophy are markers of progression towards the dementia stage in AD. More studies should address the presence of amyloid plaques [29]. Both anatomic and functional disruptions of VTA have been documented in AD; its volume reduction is related to memory issues and neuropsychiatric symptoms [30]. In addition, the disruption of VTA connectivity with other areas is responsible for agitation, irritability, and disinhibition (connectivity with cerebellar vermis and with parahippocampal gyrus). Alterations of sleep and eating patterns are associated with disruptions of VTA connectivity with the striatum and the insular cortex [30]. Our analysis of the interaction between Lobule X and VTA can also constitute an advancement of the research.

## Discussion

Some limits that could be overcome in future studies are that the matrices we used in our toy examples, “[Sec Sec9]” section, are simplified and fictitious. It would also be interesting to study and compare more real-life data to better understand the extent to which the individual impact of a disease differs from its general consequences and to distinguish more classes of *K*-operators representing different diseases. Moreover, considering the differences between male and female brains’ disease development, we could aim for a better understanding of the differences between their aging processes. Age is a risk factor for neurodegenerative diseases; further research should also address other risk factors, genetic conditions, and other kinds of data to analyse, such as EEG and MRI, in addition to the fMRI. A first step in this direction has been performed with the single case study of [4]. Unfortunately, the availability of data, especially concerning the brains of healthy volunteers, is limited.

We have several open questions. For instance, we aim to find a general theoretical property underlying the *K*-operator obtained with different computational methods that could justify the analogies between the patterns of eigenvalues that we noticed. The similarity of patterns of eigenvalues can constitute a necessary but not sufficient condition for the similarity of the original matrices. To deepen the exploration of similarities between the *K*-operators obtained from different definitions and the analysis of their action and precise biological meaning, further tests can be run, including analysis of other geometric properties. For now, we focused on some mathematical details of the *K*-operator, obtaining a “photography” of *K*, that is, a specific form of the operator for a patient, and between two time points, that is, between the fMRI data collected in two different stages of the disease. The more data information we have, the better we can approximate the action of *K* across time, that is, approximating its form as a time-dependent operator. Then, we could also define its dynamics. We also used the *K*-operator in connection with established techniques to investigate dynamics in time series, that is, RPs [24]. After a visual inspection of the *K*-operator for our case study of the AD patient from baseline to the second follow-up, we chose to focus on two brain areas, the cerebellum 10 left and the ventral tegmental area, right. We computed both the individual RPs and the joint RPs for these regions. From the observation of the patterns in the RPs and the numerical values of the RR, we noticed a progressive reduction of synchronisation of the activity between these regions over time, hinting towards a functional alteration. From the observation of the RPs in Figs. 24, 25, and 26, we noticed that the patterns in the joint RPs at the baseline are denser and more structured diagonally, than those at the first and second follow-ups, indicating a decrease of temporal coordination, and, thus, shared dynamical behaviour, between VTA and cerebellum. Looking at RPs of the individual time series, VTA presents short diagonal lines, indicating a short-term deterministic behaviour, which is progressively lost in the follow-ups, moving towards unpredictability, with the progressive disappearance of vertical or horizontal line segments. The fragmentation of patterns also occurs for the time series of the considered cerebellar area, moving towards irregularity. From the medical literature, we know the importance of VTA in the dopaminergic circuit. We also know the role of the cerebellum for motor coordination, balance, memory decline (also due to ageing, [31]), and working memory, for the latter, mostly concerning lobules VI, VII, and crus I, II [32], as well as visuospatial processes. In particular, lobule X (cerebellum 10), part of the vestibulocerebellar circuit, is also relevant for cognition [33, 34]. Thus, their progressive decoupling between VTA and cerebellum seems to be related to alterations of reward/motivation with sensorimotor and cognitive control. It can be considered a biomarker of disease progression, with apathy and impaired control. According to some authors, VTA degeneration is one of the early markers of AD [23]. Its interaction with the cerebellum is sometimes overlooked. Having focused on these regions after the analysis of the *K*-operator confirms the utility of the latter to drive new explorations in the framework of computational neuroscience.

Overall, from the pioneering intuition leading to non-invasive neuroimaging to our progressive steps with the formalisation and exploration of the *K*-operator, our article may help pave the way towards the new informative yet ethical investigation of the brain and its network-related diseases. Hence, the final objective of this research is to establish a foundational framework for the development of predictive models pertaining to the progression of neurodegenerative diseases. The mathematical representation adopted could improve the performance of predictive models by giving some prediction rules. For example, depending on the stage of the disease, it could be applied to predict the timing and severity of the disease. Neurodegenerative diseases are a class of disorders characterised by the progressive degeneration of the nervous system’s structure and function. This study aims to provide the basis for exploiting the potential inherent in integrating innovative representations of brain impairment with sophisticated machine-learning methodologies.

By leveraging AI-driven approaches, it becomes possible to automatically detect complex, nonlinear patterns in brain connectome alterations that may be subtle or difficult to capture using classical mathematical operators alone. For instance, AI models can be trained to classify different neurological disease states or progression stages based on features extracted from the *K*-operator transformations, potentially improving diagnostic accuracy and personalised prognosis. Additionally, ML techniques can be employed as preprocessing steps to identify clusters or subgroups of subjects with similar neuroimaging patterns before computing the *K*-operator. This stratification can improve the specificity of the analysis by focusing on more homogeneous cohorts, which may reveal distinct network alterations linked to different disease phenotypes or progression stages. Conversely, ML can also be applied at later stages—after computing the *K*-operator—to cluster matrices with similar spectral or structural features. Such clustering may enable the characterisation of individuals with unique or atypical disease trajectories, potentially informing personalised diagnosis and treatment strategies. By integrating these ML-driven clustering approaches, our framework gains increased flexibility and interpretability, helping to uncover latent patterns in complex neurological disorders that may not be evident through traditional analyses alone.

To provide concrete evidence when discussing integration with ML approaches in our framework, it is worth noting that machine learning techniques have already been integrated into our research framework in the study presented in [12]. Specifically, we employed a Multi-Layer Perceptron (MLP) to predict the progression of Alzheimer-Perusini’s disease by forecasting the *K*-operator based on resting-state fMRI connectivity matrices collected during the patients’ initial clinical visit. The primary objective was to evaluate the potential of the *K*-operator as a diagnostic biomarker capable of predicting disease progression. In this approach, the trained MLP model used the initial connectivity data to predict the corresponding *K*-operator for test patients, enabling the assessment of future network alterations linked to disease evolution. This combination of ML and the mathematically grounded *K*-operator illustrates the promising synergy between data-driven and theory-driven methods in enhancing diagnostic accuracy and personalised prognosis in neurodegenerative diseases.

## Limitations

Our study is overall a methodological investigation, which is fully shown for one main case. As such, it presents limitations. Our work paves the way towards more in-depth studies, using and improving the definition of the *K*-operator in conjunction with time-series analysis and established topological measures for complex networks, as centrality and modularity. Our study, with the proposed methods and results, has a methodological, proof‑of‑concept scope.

## Conclusions

In our study, we proposed a method to investigate the *K*-operator and its properties from a mathematical point of view, namely the computation of its eigenvalues and eigenvectors. We applied this to the matrix representing the compromised brain $\mathcal {G}^k$ and to the *K*-operators obtained with two different computational techniques, i.e., the element-wise multiplication and the row-by-column one. According to our results, we can find some analogies between the general matrices obtained through the two methods, above all for what concerns their eigenvalues. We applied such a technique to some real data as well, finding results similar to the theoretical ones. This leads us to think about a possible link between the two computational techniques, which could be further investigated through a perturbation-based approach, as we mentioned in “Eigenvalues and Eigenvectors” section.

Thus, summarising, we worked with three different definitions of the action of the *K*-operator, considering both simulated matrices and real data, and comparing the trend of their eigenvalues. We noticed vertical sparsity correspondence, empirically confirming the correspondence of the approaches, and thus gathering information from all of them, and getting hints on which areas are more impacted by the action of the disease. Further research can also explore the correlation between not only submatrices of the *K*-operator, the corresponding time series in the connectivity matrices, and their respective RPs. and the corresponding density-based recurrence measures, applying to neuroscience the most recent developments in physics [35].

## Data Availability

No datasets were generated or analysed during the current study.

## Footnotes

1 Obtained through https://www.wolframalpha.com/

2 https://ida.loni.usc.edu/collaboration/access/appLicense.jsp

3 In general, it is possible to quantify the degree of sparsity of matrices with: $\sigma =\frac{ |{{A_{i,j} \ne 0|}}}{n\cdot m} \ll 1.$

4 The concept is the following: given two matrices *A* and *B* with real entries, this product is defined using a kernel function, known as *k*(*x*, *y*), that computes a scalar similarity between *x* and *y*, which are vectors. The product is a matrix *K*, where $K_{i,j}=k(a_i,b_j)$. In the latter expression, we have that $a_i$ is the row-vector from the matrix *A*, $b_j$ is the column vector of the matrix *B*, and *k*, as we said, is the kernel function. This function could have several forms, but the one that we choose as our kernel is the Gaussian:

19 $$\begin{aligned} k(x,y) = e^{- \gamma \cdot \Vert x-y \Vert ^{2}}. \end{aligned}$$

5 +10 for the shift

6 +1 for the shift

## Code Availability

The code can be accessed at https://github.com/sofiafazio/K_operator_Jupyter_eigenavalues (DOI 10.5281/zenodo.14281405). For “A First Comparison of Eigenvalues for Real Data” section, see https://github.com/medusamedusa/K_operator_parkinson (DOI 10.5281/zenodo.14162649), patient B, #100006, atlas AAL3. For the computation of the recurrence plots, the code by Johann Faouzi has been adapted (https://pyts.readthedocs.io/en/latest/auto_examples/image/plot_single_rp.html), and the code for the complete $\mathcal {G}$s, complete *K*s, measures, and recurrence analysis in “An AD Case Study, Focusing on a Pair of ROIs” section can be accessed at https://github.com/medusamedusa/AAL3_K-operator_AD and https://codeberg.org/medusamedusa/AAL3_K-operator_AD-main (file ADNI_AAL3_modified_test_May_F_with_other_follow_up_january_updated.ipynb).
